# Changes in the expression of genes encoding type IV pili-associated proteins are seen when *Clostridium perfringens* is grown in liquid or on surfaces

**DOI:** 10.1186/s12864-020-6453-z

**Published:** 2020-01-14

**Authors:** Samantha R. Soncini, Andrea H. Hartman, Tara M. Gallagher, Gary J. Camper, Roderick V. Jensen, Stephen B. Melville

**Affiliations:** 1 Department of Biological Sciences, Virginia Tech, Blacksburg, VA 24061 USA; 2 Current address: UPMC Genome Center, Pittsburgh, PA USA; 3 Current address: Department of Molecular Biology & Biochemistry, University of California, Irvine, USA

**Keywords:** *Clostridium perfringens*, Gene expression profiling, Type IV pili, Motility, Translation, Adherence

## Abstract

**Background:**

*Clostridium perfringens* is a Gram-positive anaerobic pathogen that causes multiple diseases in humans and animals. *C. perfringens* lack flagella but have type IV pili (TFP) and can glide on agar surfaces. When *C. perfringens* bacteria are placed on surfaces, they become elongated, flexible and have TFP on their surface, traits not seen in liquid-grown cells. In addition, the main pilin in *C. perfringens* TFP, PilA2, undergoes differential post-translational modification when grown in liquid or on plates. To understand the mechanisms underlying these phenotypes, bacteria were grown in three types of liquid media and on agar plates with the same medium to compare gene expression using RNA-Seq.

**Results:**

Hundreds of genes were differentially expressed, including transcriptional regulatory protein-encoding genes and genes associated with TFP functions, which were higher on plates than in liquid. Transcript levels of TFP genes reflected the proportion of each protein predicted to reside in a TFP assembly complex. To measure differences in rates of translation, the *Escherichia coli* reporter gene *gusA* gene (encoding β-glucuronidase) was inserted into the chromosome downstream of TFP promoters and in-frame with the first gene of the operon. β-glucuronidase expression was then measured in cells grown in liquid or on plates. β-glucuronidase activity was proportional to mRNA levels in liquid-grown cells, but not plate-grown cells, suggesting significant levels of post-transcriptional regulation of these TFP-associated genes occurs when cells are grown on surfaces.

**Conclusions:**

This study reveals insights into how a non-flagellated pathogenic rod-shaped bacterium senses and responds to growth on surfaces, including inducing transcriptional regulators and activating multiple post-transcriptional regulatory mechanisms associated with TFP functions.

## Background

Bacteria in liquid environments use flagella-mediated swimming to facilitate their environmental lifestyle but can then switch from a planktonic lifestyle to a surface mode of existence in the form of biofilms. To make the switch, bacteria usually need to sense the presence of a surface. Some bacteria that use flagella for swimming sense a surface by detecting the inhibition of flagellar rotation, including *Vibrio parahaemolyticus*, a Gram-negative bacterium, in which surface sensing occurs by sensing inhibition of rotation of the polar flagella [1]. Another type of surface organelle associated with surface sensing is Type IV pili (TFP), which are filaments used for many functions, such as motility, adherence to surfaces (including host cells), natural transformation, and biofilm formation [2]. TFP are composed of a single protein (pilin) that is polymerized by a molecular complex embedded in the cell envelope of Gram-negative and Gram-positive bacteria [3]. Surface sensing by TFP-associated components has been observed, primarily in studies involving *P. aeruginosa*. The PilY1 protein has been shown to be involved in surface-dependent increases in virulence and it was proposed that a mechanosensing domain of the protein was important for this function [4]. Mechanosensing of shear forces by TFP and the PilY1 protein led to increased levels of cyclic-di-GMP and associated phenotypes such as biofilm formation [5]. A methyl-accepting chemotaxis-like protein, PilJ, interacts with the major pilin of *P. aeruginosa* (PilA) to regulate cAMP levels and transcriptional control of TFP and flagella genes after attachment of TFP to surfaces [6].

Even though all, or nearly all, Clostridia have TFP [3], surface sensing via TFP has not been studied in these bacteria. The pathogenic bacterium *Clostridium perfringens* represents an interesting opportunity to study surface sensing in Clostridia, since it has TFP but lacks flagella and chemotaxis systems as well as any homologs of the regulatory circuits described above [3, 7–9]. Despite a lack of flagella-mediated swimming ability, the bacteria do show phenotypic and physiological differences when grown in liquid versus plate media. *C. perfringens* exhibits gliding motility on plates in which cells line up in an end to end fashion and move away from a colony, but this motility and formation of the end to end alignment of cells does not occur in liquid cultures [3, 9]. In liquid cultures, the bacteria remain suspended in the fluid column as individual cells and are shorter in length in comparison to agar plate grown cells (4.5 ± 0.1 μm versus 6.2 ± 0.2 μm (*P* < 0.001), respectively, for *C. perfringens* strain 13, see Experimental Procedures). We also discovered that *C. perfringens* grown on agar plates adheres to mouse myoblast (C2C12) cells [10] but when grown in liquid they lose adherence to these cells (unpublished data). For this study, we were interested in measuring the expression levels of TFP-associated genes to determine if they were regulated by surface sensing mechanisms and wished to identify genes responsible for regulating these surface-dependent phenotypes.

Bacteria were grown on three different types of media, in both liquid and on plates, to identify genes expressed at higher levels on plates. Our hypothesis was that surface sensing would be independent of the metabolic state of the cells and that finding genes with higher expression on plates for all three media would allow us to identify those genes associated with, or responding to, surface sensing. We used a combination of Western blots, RNA-Seq and promoter fusions to the *gusA* gene to identify changes in pilin protein levels, as well as transcription and translation of TFP-associated genes that occur when the bacteria are grown on a surface versus liquid media. We found that in media with higher amounts of glucose, several TFP genes were transcribed at higher levels on plates than in liquid. We also found that there is a significant amount of post-transcriptional regulation of TFP genes on plates but not in liquid, suggesting additional TFP regulatory systems are recruited when the cells are grown on a surface. RNA-Seq also allowed us to identify multiple promoters and terminators within the main TFP locus that act in a highly coordinated manner to produce the proper stoichiometry of TFP proteins needed for TFP assembly and retraction. Finally, analysis of all genes with higher expression on plates points to a putative SigV/anti-SigV protein pair that may play a role, still not clearly defined, in adapting to growth on surfaces.

## Results

### PilA2 is the major pilin needed for adherence to mouse myoblasts

**Fig. 1:**
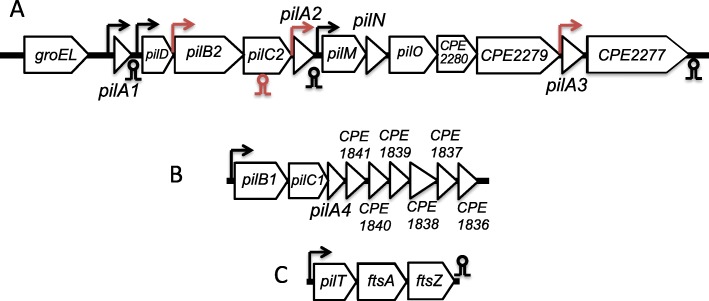
**a** Operon map of the major TFP locus in strain 13. **b** Operon map of the minor TFP operon in strain 13. **c** *pilT*, the retraction ATPase necessary for TFP-mediated motility, lies in an operon with cell-division genes *ftsA* and *ftsZ*. Arrows indicate the location of promoters, circles and stems denote rho-independent terminators. Elements in red indicate new regulatory features identified by RNA-Seq. Gene sizes not to scale

**Fig. 2:**
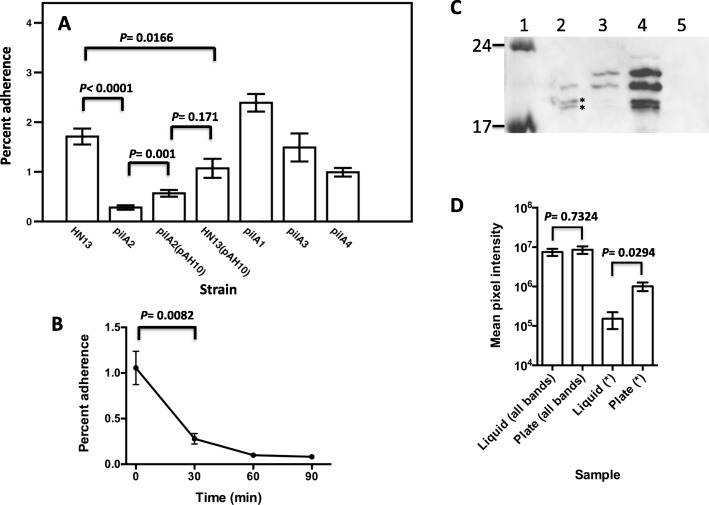
**a** Adherence of pilin mutant strains of *C. perfringens* to C2C12 myoblasts. *pilA1* mutant, strain AH7; *pilA2* mutant, strain AH8; *pilA3* mutant, strain AH9; *pilA4* mutant, strain AH10. pAH10 carries a copy of the *pilA2* gene under control of a lactose-inducible promoter (5 mM lactose was used to induce *pilA2* expression). The mean and SEM of at least five replicates each from at least three independent samples are shown; the *P* values shown were calculated using the two-tailed students t-test. **b** Time course showing changes in adherence of strain HN13 to C2C12 myoblasts after transfer from BHI plates to BHI liquid. The mean and SEM of at least five replicates each from two independent samples are shown; the *P* value shown was calculated using the two-tailed students t-test. **c** Representative anti-PilA2 Western blots. Lane 1, molecular weight markers with sizes (kDa) indicated on the left; lane 2, strain HN13 grown on BHI plates; lane 3, strain HN13 grown in BHI liquid; lane 4, strain AH8 (Δ*pilA2*) with pAH10 grown on BHI plates; lane 5, strain AH8 (Δ*pilA2*) grown on BHI plates with 5 mM lactose. Asterisks denote bands used for densitometry measurements shown in panel D. **d** Densitometry on four bands identified as specific to PilA2 in Western blots. Images from five individual Western blots were analyzed, and the mean and SEM are shown. “All bands” denotes the total mean pixel intensity for the four bands for each sample that can be most clearly seen in lane 4. The asterisks denote the total mean pixel intensity for the two lower bands visible in panel C that were marked with asterisks

We have shown in a previous report that *C. perfringens* strain 13 can adhere to C2C12 mouse myoblasts [10]. The TFP retraction ATPase PilT was shown to be necessary for efficient adherence to C2C12 cells [10]. *C. perfringens* strain 13 encodes four pilin proteins, PilA1, PilA2, PilA3, and PilA4, and the genes are located in different TFP-associated loci (Fig. 1). To determine which pilins are required for adherence to C2C12 myoblasts, we made in-frame deletions of the genes encoding each pilin and performed adherence assays on the mutants and wild type (WT) strain using bacteria grown on BHI agar plates (Fig. 2a). Of the four pilins, deleting the *pilA2* gene resulted in the most significant loss in adherence (84%), *pilA4* deletion decreased adherence by 42%, and *pilA1* and *pilA3* deletions had no effect (Fig. 2a). Complementation of the *pilA2* deletion strain with a plasmid carrying a wild-type copy of the gene (pAH10) partially restored adherence, while expression of the *pilA2* gene in the wild-type strain HN13 (i.e., containing both a chromosomal copy and plasmid-borne copy of *pilA2*) led to significantly decreased adherence, suggesting high levels of expression of PilA2 interfere with normal levels of adherence (Fig. 2a).

While carrying out the adherence assays, we noticed that WT cells grown in liquid BHI adhered poorly to C2C12 cells (0.05 ± 0.015% adherence). To determine the kinetics of the change in binding adherence between plate grown and liquid grown cells, bacteria were scraped off BHI plates and suspended in BHI liquid, and the levels of adherence to C2C12 cells was measured over a 90-min time frame. Eighty percent of the binding capacity was lost after 30 min and 90% after 90 min in liquid (Fig. 2b), suggesting that a shift from plates to liquid lowers adherence to C2C12 cells by altering the level of TFP on the surface of cells.

### PilA2 undergoes differential post-translational modifications when cells are grown on plates or in liquid BHI

Since PilA2 was necessary for the large majority of adherence (Fig. 2a), we measured the levels of PilA2 in the cytoplasmic membranes of the WT, Δ*pilA2* mutant (strain AH8), and complemented strains in plate and liquid-grown cells using anti-PilA2 antibodies in Western blots (Fig. 2c). Membranes prepared from cells grown on BHI plates showed the presence of bands corresponding to PilA2 with molecular weights of 18 and 19 kDa as well as two additional bands at molecular weights of 21 and 22.5 kDa, but at lower levels (Fig. 2c, lane 2). Based on the predicted molecular weight of the mature PilA2 protein (18.1 kDa), the 18 and 19 kDa bands likely represent the proteolytically processed and unprocessed form of PilA2 due to the activity of the pre-pilin peptidase (PilD), respectively. In cultures grown in liquid BHI, the 18 and 19 kDa forms were visible at significantly lower levels than those of the 21 and 22.5 kDa forms (Fig. 2c, lane 3). Expression of the *pilA2* gene from a lactose-inducible promoter on a plasmid in the *pilA2* mutant strain showed the presence of all four bands at much higher intensity than the WT strain (Fig. 2c, lane 4). As a control, membranes from the *pilA2* mutant strain showed no PilA2-specific bands (Fig. 2c, lane 5). The nature of the two higher molecular weight forms of PilA2 is unknown, however, due to their increased mass, they likely represent post-translational covalent modifications of PilA2. The *pilA2* gene expressed on the lactose-inducible promoter did not have any additional coding sequences present other than the *pilA2* gene itself. Since the four bands visible when *pilA2* was expressed from a plasmid in a *pilA2* deletion strain (lane 4) match the sizes of those in the WT strain, this makes it unlikely that there are alternative start sites for the *pilA2* translation that could account for the larger forms seen in lanes 2 and 3. Densitometry on the PilA2 Western blots to measure the relative proportions of the different PilA2 forms showed the levels of all four bands added together was the same between liquid grown and plate grown cells, but the levels of the 18 and 19 kDa forms (i.e., the unmodified versions) were twice as high in plate-grown cells than in liquid-grown cells (Fig. 2d), suggesting post-translational modification occurs at higher levels in liquid-grown cells.

### A comparison of transcript levels using RNA-Seq shows significant changes in hundreds of genes in plates vs liquid

**Fig. 3:**
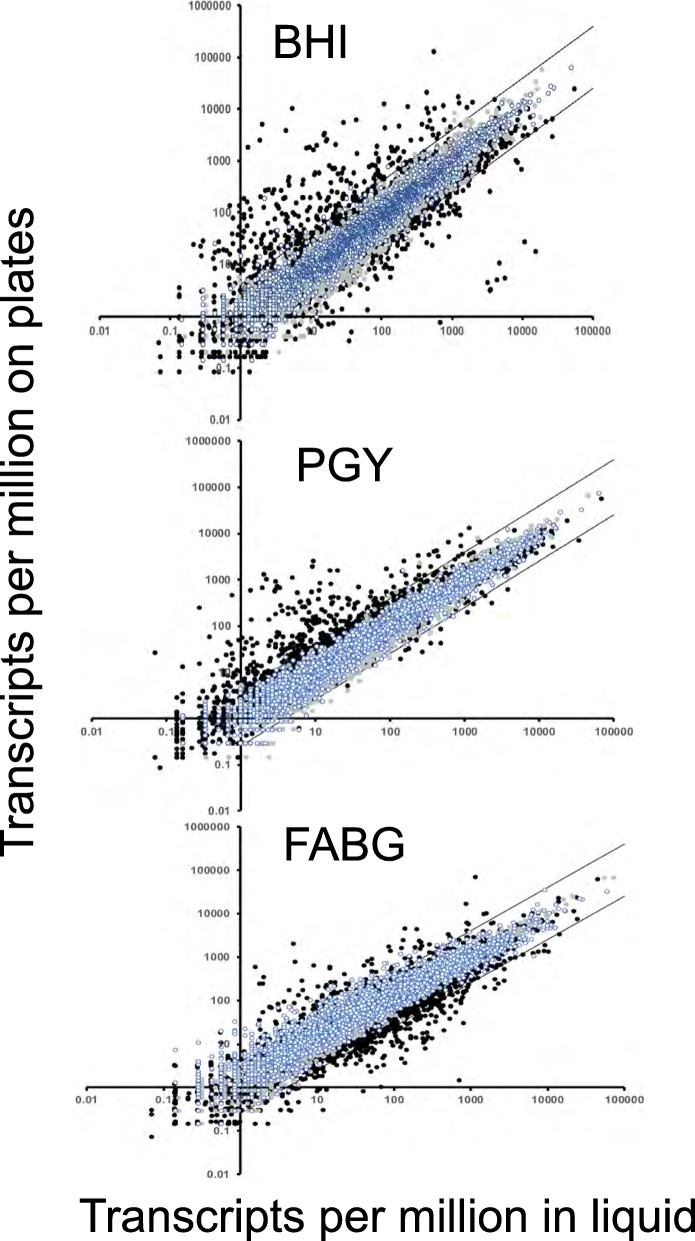
Plot showing the TPM on plates versus liquid grown cells from RNA-Seq. Two types of data are presented in each plot, the first are comparisons of replicate samples in liquid (blue-white circles) and on plates (gray circles). Note that the replicates rarely diverged from the four-fold range indicated by the parallel bars. The second type is represented by black circles, which showing the mean and SEM of duplicate samples plotted for each gene in plate versus liquid axes. Black circles that lie outside of the bars delineate genes that are regulated > 4-fold between the different conditions for each medium

While PilA2 total protein levels were similar in cells grown on BHI plates or liquid (Fig. 2c and d), we lacked information about the levels of other TFP proteins in plate-grown versus liquid grown cells. We also hoped to identify potential transcriptional regulators that may affect transcript levels of TFP-associated genes. Therefore, we used RNA-Seq to measure the transcript levels of the genes in *C. perfringens* strain HN13 grown, in duplicate, in three different types of liquid media (BHI, PGY, and FABG) along with the corresponding plates made with the same media. RNA was extracted from the cells and used for RNA-Seq. An FDR (q value) of < 0.05 and a differential gene expression of log_2_ > 2 in expression level were used as cutoffs to represent significant differences. Comparisons of plate versus liquid-grown cells showed that there were hundreds of differences in the expression of genes in each of the three different types of media (Fig. 3). The number of differentially expressed genes across all samples are listed in Additional file 1: Table S4 and in Additional file 2: Table S5 is listed the comparisons for each individual gene. The 135 genes that showed higher expression on plates versus liquid as well as the 23 genes with higher expression in liquid versus plates for all media are listed in Table 1. The majority of the genes that were expressed higher on plates in all three media are involved in utilization of carbon sources, including proteins involved in an arginine deiminase fermentation pathway and ethanolamine utilization (Table 1). There were four genes that encoded identifiable transcriptional regulatory proteins, *argR*, *purR*, *hipB*, and *nagC*. Each of these genes is located in an operon related to specific metabolic functions: *argR*, encoding the arginine repressor in an operon with genes encoding enzymes for the arginine deiminase pathway, *purR* in an operon encoding an ABC transporter of spermidine/putrescine, *hipB* in an operon encoding an ABC transporter for ribose, and *nagC* in an operon with genes encoding an alpha-glucosidase and ABC transporter for sugars [11], suggesting none of these are pleiotropic regulators for growth on surfaces. However, another gene involved in transcriptional regulation, the gene encoding CPE0560, which encodes a putative membrane bound anti-SigV protein, was also expressed higher on plates (Table 1), although its cognate, the gene encoding SigV, was not. Although CPE0560 has little sequence identity to other anti-SigV proteins, we predicted it to have this function based on its synteny (immediately 5′ to *sigV*) and membrane topology, which is nearly identical to that of the anti-SigV protein from *B. subtilis*, RsiV [12]. The role of the SigV/anti-SigV proteins in bacterial morphology was investigated by making deletions in each gene.

**Table 1:** Genes differentially trnascribed on plates or in liquid

Gene Name	Gene Product	Locus tag	DGE (LFC)^a^	FDR (q value) ^a^
Genes transcribed at higher levels on plates than liquid				
*fus*	Elongation factor G	CPE0079	2.010	0.0276
*mdh*	Alcohol dehydrogenase	CPE0085	5.323	0.0405
*iolB*	Myo-inositol catabolism protein	CPE0088	5.361	0.0276
*iolD*	Myo-inositol catabolism protein	CPE0089	5.840	0.0276
*mviM*	Probable dehydrogenase	CPE0090	6.458	0.0405
*iolE*	Myo-inositol catabolism protein	CPE0091	6.367	0.0405
	Na+/myo-inositol cotransporter	CPE0092	6.517	0.0405
*mviM*	Probable dehydrogenase	CPE0093	6.825	0.0405
*caiD*	Crotonase	CPE0095	6.801	0.0405
	Propionate CoA-transferase	CPE0096	6.666	0.0405
*acdS*	Acyl-CoA dehydrogenase	CPE0097	6.615	0.0276
*gldA*	Glycerol dehydrogenase	CPE0099	3.575	0.0405
*ldh*	L-lactate dehydrogenase	CPE0103	2.347	0.0276
	Hypothetical protein	CPE0105	2.200	0.0276
	Hypothetical protein	CPE0113	4.346	0.0405
	Probable transposase	CPE0139	2.209	0.0405
*rbsK*	2-keto-3-deoxygluconate kinase	CPE0146	3.584	0.0405
*bglR*	Beta-glucuronidase	CPE0147	2.973	0.0276
*fabG*	Probable oxidoreductase	CPE0149	4.098	0.0276
*eda*	KDPG/KHG aldolase	CPE0150	4.529	0.0276
*uxuA*	D-mannonate dehydrolase	CPE0151	4.598	0.0276
*uxaC*	Glucuronate isomerase	CPE0152	4.107	0.0276
*uidB*	Glucuronide permease	CPE0153	3.929	0.0508
*bglX*	Probable beta-hexosamidase A	CPE0154	3.756	0.0276
*potE*	Membrane-spanning transporter protein	CPE0166	5.109	0.0276
*pbg*	Beta-galactosidase	CPE0167	6.142	0.0276
*arcA*	Arginine deiminase	CPE0168	8.558	0.0276
*arcB*	Ornithine carbamoyl transferase	CPE0169	9.532	0.0276
*arcD*	Arginine ornithine antiporter	CPE0170	9.285	0.0405
*arcC*	Carbamate kinase	CPE0171	10.053	0.0276
*argR*	Arginine repressor	CPE0172	9.292	0.0276
*ackA*	Acetate kinase	CPE0217	2.473	0.0276
*galE*	UDP-glucose 4-epimerase	CPE0286	2.507	0.0276
*lldP*	Probable lactate permease	CPE0310	4.960	0.0405
*etfB*	Electron transfer flavoprotein beta-subunit	CPE0311	5.305	0.0405
*etfA*	Electron transfer flavoprotein alpha-subunit	CPE0312	5.131	0.0405
*glcD*	Probable glycolate oxidase subunit	CPE0313	5.530	0.0405
*fucK*	Rhamnulokinase	CPE0317	4.832	0.0405
*fucI*	L-fucose isomerase	CPE0318	5.490	0.0405
*fucA*	L-fuculose-phosphate aldolase	CPE0319	5.211	0.0405
*manX*	Phosphotransferase system, mannose/fructose-specific component IIA	CPE0320	5.914	0.0405
*manY*	Probable PTS system	CPE0322	5.990	0.0405
*manZ*	Probable PTS system	CPE0323	5.472	0.0276
	Probable glycosyl hydrolase	CPE0324	4.220	0.0405
	Hypothetical protein	CPE0325	4.582	0.0276
*lacA*	Galactose-6-phosphate isomerase	CPE0326	3.756	0.0276
*lacB*	Galactose-6-phosphate isomerase	CPE0327	4.059	0.0405
	Hypothetical protein	CPE0329	3.447	0.0405
*mgtA*	Probable cation-transporting ATPase	CPE0333	2.606	0.0276
*ugpB*	ABC-type sugar transport system, periplasmic component	CPE0371	3.351	0.0405
*ugpA*	Probable ABC transporter	CPE0372	3.083	0.0405
*ugpE*	Probable ABC transporter	CPE0373	3.126	0.0405
*aga*	Alpha-galactosidase	CPE0374	3.251	0.0276
*skn1*	Endo-beta-galactosidase C	CPE0375	3.511	0.0276
*arcD*	Probable amino acid permease	CPE0389	3.325	0.0276
*dchS*	Histidine decarboxylase	CPE0390	3.404	0.0276
	Conserved hypothetical protein, six-hairpin glycosidase	CPE0426	3.005	0.0276
*dedA*	Alkaline phosphatase-like protein	CPE0455	4.214	0.0405
	Conserved hypothetical protein	CPE0456	5.954	0.0405
*nanJ*	Exo-alpha-sialidase	CPE0553	3.651	0.0405
	Hypothetical protein	CPE0554	4.438	0.0405
	Hypothetical protein, in sigV operon	CPE0560	3.806	0.0405
*ribB*	Riboflavin biosynthesis protein	CPE0566	4.731	0.0405
*pflA*	Pyruvate-formate lyase-activating enzyme	CPE0660	2.014	0.0276
	Hypothetical protein	CPE0669	2.475	0.0276
*nanI*	Exo-alpha-sialidase	CPE0725	2.373	0.0276
*lipB*	Probable lipase	CPE0742	2.368	0.0276
*cstA*	Probable carbon starvation protein (peptide utilization)	CPE0743	2.902	0.0276
*fepG*	Probable iron (III) dicitrate ABC transporter	CPE0794	2.994	0.0405
	Conserved hypothetical protein	CPE0806	2.526	0.0276
	Hypothetical protein	CPE0808	2.776	0.0276
	Endo-beta-N-acetylglucosaminidase	CPE0818	4.763	0.0405
*rubY*	Rubrerythrin	CPE0855	5.144	0.0276
*ams1*	Alpha-mannosidase	CPE0856	2.716	0.0276
*gntT*	Probable gluconate permease	CPE0860	2.095	0.0498
	Hypothetical protein	CPE0863	2.281	0.0276
	Hypothetical protein	CPE0876	2.380	0.0276
*pduL*	Probable propanediol utilization protein	CPE0905	4.429	0.0276
*nrdD*	Probable anaerobic ribonucleotide reductase	CPE0917	3.741	0.0276
	Hypothetical protein	CPE0918	3.713	0.0405
	Hypothetical protein	CPE0919	2.303	0.0405
*pduC*	Coenzyme B12-dependent glycerol dehydrogenase large subunit	CPE0929	4.916	0.0405
	Coenzyme B12-dependent glycerol dehydrogenase medium subunit	CPE0930	5.177	0.0276
*pduE*	Coenzyme B12-dependent glycerol dehydrogenase small subunit	CPE0931	4.927	0.0276
	Probable glycerol dehydratase large subunit	CPE0932	5.155	0.0276
	Conserved hypothetical protein	CPE0934	4.864	0.0405
	Hypothetical protein	CPE0982	3.345	0.0276
*sdhB*	L-serine dehydratase beta subunit	CPE0988	2.614	0.0405
*sdhA*	L-serine dehydratase alpha subunit	CPE0989	2.606	0.0276
*tdcF*	Probable translation initiation inhibitor	CPE1012	2.954	0.0405
*thd*	Threonine dehydratase	CPE1165	2.659	0.0276
*gltP*	Probable glutamate/ aspartate transporter	CPE1167	4.116	0.0276
*pfk*	6-phosphofructokinase	CPE1185	2.492	0.0276
*ugpB*	ABC-type sugar transport system, periplasmic component	CPE1257	5.358	0.0405
*eno*	Enolase	CPE1299	2.171	0.0405
*galM*	Aldose 1-epimerase	CPE1344	3.795	0.0276
*galK*	Galactokinase	CPE1345	3.935	0.0405
*galT*	Galactose-1-phosphate-uridyl transferase	CPE1346	5.420	0.0405
*ams1*	Alpha-mannosidase	CPE1415	2.732	0.0276
*clpB*	ATPase with chaperonin activity	CPE1428	2.641	0.0276
	Uncharacterized membrane protein, peptidase	CPE1452	2.064	0.0276
	Hypothetical protein	CPE1592	2.340	0.0276
*purR*	Probable transcriptional regulator, LacI/PurR family	CPE1626	3.087	0.0276
*rbsB*	Probable ribose ABC transporter	CPE1627	3.103	0.0276
*rbsC*	Probable ribose ABC transporter	CPE1629	2.619	0.0405
*rbsA*	Probable ribose ABC transporter	CPE1630	2.403	0.0276
*trmU*	Probable tRNA (5-methylaminomethyl-2-thiouridylate)-methyltransferase	CPE1783	2.530	0.0276
*nifU*	Probable nitrogen fixation protein	CPE1784	2.309	0.0276
*dacF*	Serine-type D-Ala-D-Ala carboxypeptidase	CPE1806	2.596	0.0276
*deoA*	Pyrimidine-nucleoside phosphorylase	CPE1807	2.672	0.0276
	Alpha-L-fucosidase	CPE1876	3.336	0.0405
*hipB*	Predicted transcriptional regulator	CPE1967	3.060	0.0405
*potB*	Probable spermidine/putrescine ABC transporter	CPE1969	2.143	0.0405
*pldB*	Lysophospholipase	CPE1989	2.345	0.0276
*podK*	Pyruvate phosphate dikinase	CPE2011	2.691	0.0276
*potE*	Probable glutamate gamma-aminobutyrate antiporter	CPE2060	2.994	0.0405
	Alpha-glucosidase	CPE2076	2.114	0.0405
*nagC*	Probable transcriptional regulator	CPE2077	2.213	0.0405
*ugpB*	ABC-type sugar transport system, periplasmic component	CPE2078	3.148	0.0405
*ugpE*	Probable ABC transporter	CPE2081	3.132	0.0405
*lplB*	Probable ABC transporter	CPE2082	3.087	0.0405
*glnQ*	Probable amino acid ABC transporter	CPE2092	2.263	0.0276
	Hypothetical protein	CPE2100	2.371	0.0276
	Probable mercuric ion-binding protein	CPE2151	2.128	0.0276
*lraI*	Metal transport and potential adhesin	CPE2158	2.531	0.0276
*nlpD*	Membrane protein related to metalloendopeptidase	CPE2182	2.322	0.0480
*lepW*	Signal peptidase type I	CPE2295	2.761	0.0405
*add*	Probable adenosine deaminase	CPE2506	3.538	0.0276
	Conserved hypothetical protein with CXXC pairs	CPE2549	4.434	0.0276
*lpd*	Probable oxidoreductase	CPE2550	3.875	0.0276
*glpA*	Glycerol-3-phosphate dehydrogenase	CPE2551	2.652	0.0276
*glpP*	Probable glycerol uptake operon antiterminator	CPE2553	2.101	0.0276
*agaS*	Probable tagatose-6-phosphate aldose/ketose isomerase	CPE2625	2.114	0.0405
*gatY*	Probable tagatose-bisphosphate aldolase	CPE2626	2.311	0.0276
*manY*	Probable PTS system	CPE2631	2.527	0.0405
Genes transcribed at higher levels in liquid than on plates				
	Predicted membrane protein in *amiC* operon	CPE0117	2.138	0.0276
*mscL*	Large-conductance mechanosensitive channel	CPE0174	2.349	0.0276
*adh*	Alcohol dehydrogenase	CPE0449	3.342	0.0276
*opuBA*	Glycine betaine/carnitine/choline ABC transporter	CPE0557	3.320	0.0276
*hisJ*	Amino acid ABC transporter	CPE0600	2.705	0.0502
*hisM*	Amino acid ABC transporter	CPE0601	2.959	0.0405
*glnQ*	Amino acid ABC transporter	CPE0602	3.759	0.0276
*argG*	Argininosuccinate synthase	CPE0691	3.140	0.0405
	Hypothetical protein, alone in operon	CPE0768	2.107	0.0276
*pfs*	5′-methylthioadenosine/S-adenosylhomocysteine nuclosidase	CPE1050	4.068	0.0276
*cfa*	Cyclopropane-fatty-acyl-phospholipid synthase	CPE1051	2.711	0.0276
*sseA*	Thiosulfate sulfur transferase	CPE1052	2.314	0.0276
*adeC*	Adenine deaminase	CPE1268	2.032	0.0276
*potD*	Spermidine/putrescine-binding protein	CPE1269	2.464	0.0276
*nupC*	Pyrimidine nucleoside transporter	CPE1284	3.637	0.0276
	Hypothetical protein, alone in operon	CPE1539	3.105	0.0276
	Conserved hypothetical protein	CPE1655	2.296	0.0405
	Probable xanthine/uracil/vitamin C permease	CPE1751	2.101	0.0276
*abrB*	Stage V sporulation protein T, transcritional regulator	CPE2482	3.013	0.0276
*adhE*	Aldehyde-alcohol dehydrogenase E	CPE2531	2.187	0.0276
*spmB*	Spore maturation protein B	CPE2532	2.263	0.0405
*glnA*	Glutamine synthetase	CPE2569	2.216	0.0405
	Conserved hypothetical protein, alone in operon	CPE2585	3.485	0.0276

^a^. DGE (LFC), differential gene expression (log fold change). Calculated from the TPM from 6 samples grown on plates versus 6 samples grown in liquid

In liquid, just a single gene encoding a transcriptional regulator, *abrB*, was expressed at higher levels (Table 1). AbrB is a global transcriptional regulator that has been shown to be involved in the regulation of sporulation [13] and biofilm formation [14] in *C. perfringens*. It seems likely that differential expression of *abrB* in liquid may be due to differences in the nutritional state of the cells, since the CodY protein, which responds to nutritional signals, is a regulator of *abrB* expression in other strains of *C. perfringens* [13].

Of the 23 genes that were expressed higher in liquid media, most were involved in metabolic processes with the exception being the *mscL* gene, which encodes a large-conductance mechanosensitive channel (Table 1). Genes encoding hypothetical proteins were expressed higher on plates and in liquid, but no specific functions could be assigned to them.

Genes encoding toxins thought to be important in virulence showed highly variable regulation in liquid versus plate-grown cells (Table 2). Phospholipase c (*plc*) expression did not vary much between liquid and plates in BHI and PGY but was expressed 10-fold higher in liquid FABG than FABG plates. The *pfoA* gene, encoding perfringolysin O, was expressed 6-fold higher on BHI plates but was expressed ~ 4-fold lower on FABG plates. The genes encoding collagenase and alpha-clostripain showed a pattern similar to that of *plc*, little change on BHI and PGY but was expressed at lower levels in FABG liquid. The *nanI* gene, encoding the NanI sialidase was expressed at higher levels on plates in all three media. Except on BHI plates, the *nanJ* gene showed very low levels of expression, but was expressed at higher levels on plates in all three different types of media (Table 2). These toxin-encoding genes are subject to complex regulatory mechanisms [15] but clearly show a transcriptional response to the environment in which the bacteria are grown.

**Table 2:** Toxin gene transcript differential regulation in liquid and on plates

Toxin	BHI LIQUID AVG	BHI PLATE AVG	Ratio BHI PL:BHI LI	PGY LIQUID AVG	PGY PLATE AVG	Ratio PGY PL:PGY LI	FABG LIQUID AVG	FABG PLATE AVG	Ratio FABG PL;FABG LI
PLC	871.3^a^	998.8	1.1	1086.5	721.4	0.7	1180.0	117.4	0.1
PFO	749.5	4589.8	6.1	720.9	1181.8	1.6	417.2	115.0	0.3
Collagenase	860.5	1061.0	1.2	1656.3	1624.6	1.0	2154.4	307.7	0.1
Clostripain	1253.3	1122.2	0.9	1700.3	1126.9	0.7	1409.3	454.6	0.3
NanI	5.3	36.3	6.9	5.1	31.0	6.1	6.5	19.5	3.0
NanJ	0.3	7.1	28.4	0.2	2.3	15.0	0.4	0.7	1.8

^a^. Average transcripts per million reads for two samples

*PL* Plates, *LI* Liquid

### qRT-PCR results validate the levels of expression seen with RNA-Seq

Using the RNA-Seq TPM values obtained from the Geneious software, five TFP-related genes were chosen for qRT-PCR validation based on their stable expression in all of the tested conditions. These genes were chosen to validate the fold changes between liquid and plate cultures in the three different media tested by RNA-Seq using a second set of independent RNA samples. The pilin genes chosen were *pilA2*, *pilB2*, *pilC2*, *pilT*, and the gene encoding CPE2277. The control housekeeping gene *lon* was chosen due to its high levels of expression in the samples and its minimal change in transcription levels between all liquid and plate samples.

qRT-PCR fold changes were determined using the ΔΔC_t_ method to calculate expression fold change ranges based on the standard deviation of the qRT-PCR thresholds obtained in triplicate experiments. When fold changes were compared between the RNA-Seq TPM values and qRT-PCR values, only five of the eighteen calculated RNA-Seq fold changes did not fall in the qRT-PCR fold change ranges (Table 3). However, those five changes did show a similar trend to the qRT-PCR data, indicating that transcriptional regulatory relationships were still satisfied by the data. Therefore, the RNA-Seq dataset was supported by the qRT-PCR validation, allowing further bioinformatic conclusions to be drawn from the full data set.

**Table 3:** qRT-PCR of specific pilin-associated genes to validate RNA-Seq analyses

	RNA-Seq fold changes			qRT-PCR ΔΔC_t_ fold changes		
	BHI PL/LI	PGY PL/LI	FABG PL/LI	BHI PL/LI	PGY PL/LI	FABG PL/LI
*pilA2 (cpe2284)*	0.873*	1.326	2.339	1.21–1.69	1.07–2.05	1.33–2.93
*pilB2 (cpe2286)*	1.143	2.950	5.233	0.74–1.44	1.07–3.62	2.98–5.68
*pilC2 (cpe2285)*	0.807	3.063	4.178*	0.79–2.29	1.95–3.41	1.52–3.02
*pilT (cpe1767)*	0.614*	1.735	0.942	0.77–1.28	1.27–2.09	0.87–1.64
*cpe2277*	0.679*	3.128	1.699*	0.72–1.87	1.82–3.18	0.88–1.61
*lon (cpe2635)*	0.795	0.939	1.130			

*indicate a value out of fold change range but shows similar trends

### Transcript levels in the large pilin locus show different levels of expression between plates and liquid in PGY and FABG

**Fig. 4:**
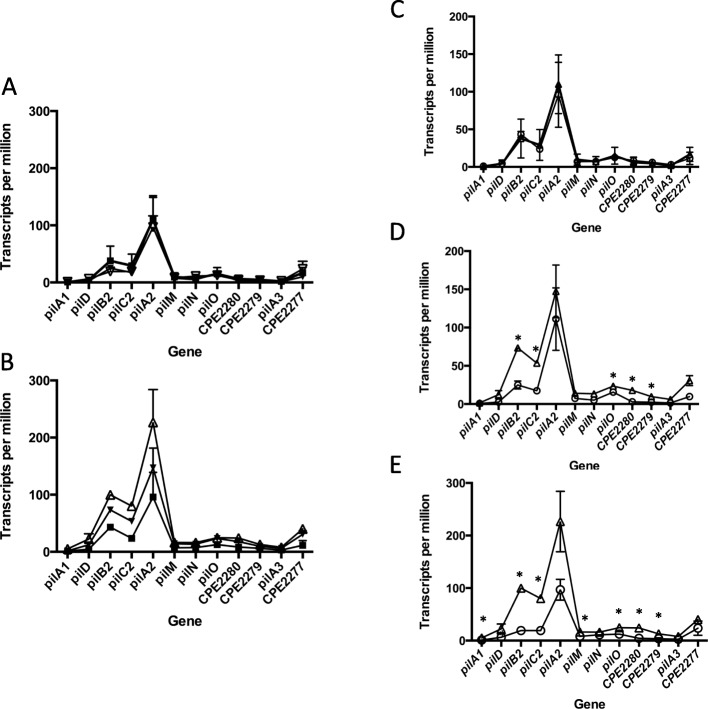
Transcript levels for genes in the large TFP operon under different conditions and media. **a** and **b** Transcript levels of cells grown in BHI (squares), PGY (inverted triangles) and FABG (open triangles) in liquid (**a**) and on plates (**b**). The mean and SEM are shown. **c**, **d**, and **e** Transcript levels in cells grown on plates (triangles) and in liquid (circles) in BHI (**c**), PGY (**d**), and FABG (**e**). The mean and SEM are shown for two independent RNA samples. Asterisks denote genes showing a significantly greater level (*P* < 0.05) of transcripts on plate versus liquid grown cells using the two-tailed students t-test. Data points were connected by lines to illustrate trends in changes of transcript levels

We examined the transcript levels of the genes in the large pilin locus extending from *pilA1* to the gene encoding CPE2277 grown on three types of plates and liquid media. In liquid cultures, there was no difference between the three different types of media (Fig. 4a). For cells grown on plates, overall expression levels were FABG>PGY > BHI (Fig. 4b). A comparison of plate versus liquid grown cells in each media showed that cells in BHI had the same levels of expression (Fig. 4c), cells in PGY had higher levels on plates versus liquid for *pilB2*, *pilC2*, *pilO*, and the gene encoding CPE2280 and CPE2279 (Fig. 4d), while bacteria in FABG had higher levels of expression on plates for *pilA1*, *pilB2*, *pilC2*, *pilM*, *pilO*, and the gene encoding CPE2280 (Fig. 4e). Under all conditions, the *pilA2* gene showed the highest level of expression followed by *pilB2* and then the *pilC2* gene.

### There is a promoter upstream of the *pilB2* gene and an intragenic terminator in the *pilC2* gene

**Fig. 5:**
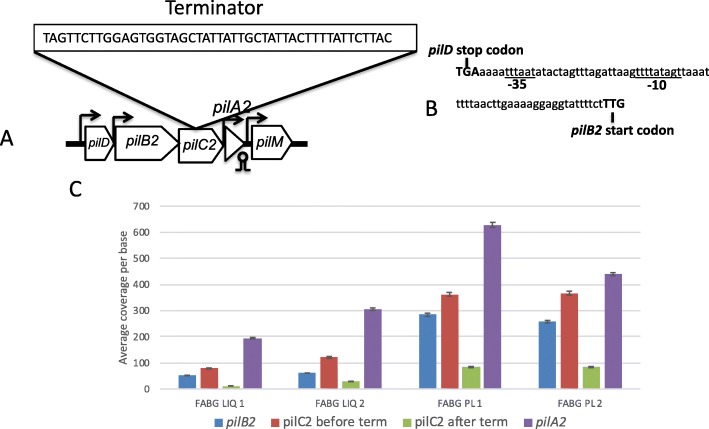
**a.** Schematic diagram showing the locations of promoters and terminators in five genes located in the large pilin operon. The sequence of a putative rho-independent terminator internal to the *pilC2* gene is shown in the box. **b.** Location of a putative promoter, designated by the −10 and − 35 notations, located between the *pilD* and *pilB2* genes. **c.** Transcript coverage per base of the *pilC2* gene and flanking genes for bacteria grown in FABG. Note the drop in transcripts after the terminator (abbreviated “term”) in *pilC2*. Similar results were seen for bacteria grown in BHI and PGY (data not shown)

A promoter has been predicted to be located upstream of the *pilD* gene [3, 9, 14, 16], and this is supported by the increased transcript levels between the *pilA1* and *pilD* genes (Fig. 4). However, the increase in transcript levels between the *pilD* and *pilB2* genes (Fig. 4) indicated an additional promoter may be present. The promoter prediction software BPROM (located online at http://www.softberry.com/) predicted a promoter is located in the intergenic region between the *pilD* and *pilB2* genes (Fig. 5a and b). Increased levels of transcripts initiating just after the promoter can be seen in Additional file 3: Figure S1.

We also noted a decrease in the transcript levels between the *pilB2* and *pilC2* genes (Fig. 4). Analysis of the *pilC2* transcript levels in coverage maps indicated a sharp drop in transcripts near the center of the gene as if a terminator is present (Additional file 3: Figure S2). The terminator prediction software ARNold (located at http://rna.igmors.u-psud.fr/toolbox/arnold/index.php) predicted that a terminator was present at the precise location where the transcript levels dropped (Fig. 5a and Additional file 3: Figure S2). Measurements of *pilC2* transcripts before and after the terminator indicate a 4–5-fold drop in transcripts occurred after the suspected terminator in all of the media and conditions tested (Fig. 5c). Terminated transcripts lacking a stop codon result in formation of a “non-stop” translation complex, which can be lethal if allowed to accumulate [17]. The large majority of bacteria use a combination of a transfer-messenger RNA (tmRNA) and a small protein, SmpB, to release the ribosome and degrade the nascent peptide and mRNA [18]. *C. perfringens* has genes encoding the tmRNA and SmpB [7], but this process has, to our knowledge, not been studied in Clostridia. So, we wanted to determine if this truncated form of PilC2 protein was present in the cells. Therefore, we expressed a version of the *pilC2* gene with 6 His codons on the C-terminus from a lactose inducible promoter in plasmid pKRAH1 [19] in *C. perfringens*. We then performed Western blots on whole cell extracts with antibodies directed against a peptide in the N-terminal domain of the PilC2 protein (residues 9–22, INSEGQREIGSQSAC) and the His_6_ tag. If present, the truncated protein would be detected by the anti-PilC2 antibodies but not the anti-His6 antibodies. However, only the full length PilC2 was detected with both antibody types (Additional file 3: Figure S3), suggesting the truncated PilC2 was being degraded, likely by a tmRNA-SmpB-dependent mechanism.

### Gaps in RNA-Seq results indicate likely transcript start sites upstream of the *pilA2* and *pilA3* genes

**Fig. 6:**
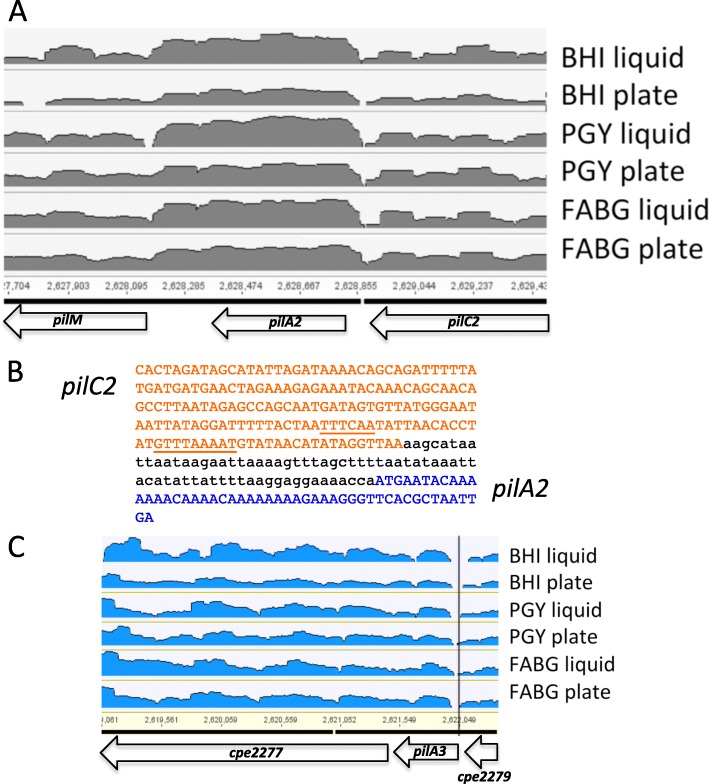
**a** Depth of coverage (volcano) plot (log scale) showing the transcript levels covering the *pilA2* gene for cells grown under the indicated conditions. **b** The location of a putative promoter for *pilA2* (underline) located in the 3′ end of the *pilC2* gene. The coding region for *pilC2* is shown in red, the coding region for *pilA2* in blue. **c** Location of a gap in transcripts between the gene encoding CPE2279 and *pilA3* genes (vertical black line)

Analysis of the RNA-Seq data from the current studies, in the form of volcano plots, indicated there was a gap in the transcripts immediately upstream of the *pilA2* gene (Fig. 6a). The gap could represent transcription terminating and restarting or an RNA processing site; the latter has been suggested for the region upstream of the *pilA2* gene [14, 16]. One potential promoter for the *pilA2* gene in this region was identified by the BPROM software (http://www.softberry.com/) in the DNA encoding the C-terminal domain of the *pilC2* gene (Fig. 6b). Examination of transcript levels at the junction of the gene encoding CPE2279 and *pilA3* genes showed the presence of a gap in transcription, suggesting there may also be a promoter specific to the *pilA3* gene (Fig. 6c).

### The *pilB1* operon is transcribed at low levels and likely consists of nine genes instead of the four predicted in initial studies

**Fig. 7:**
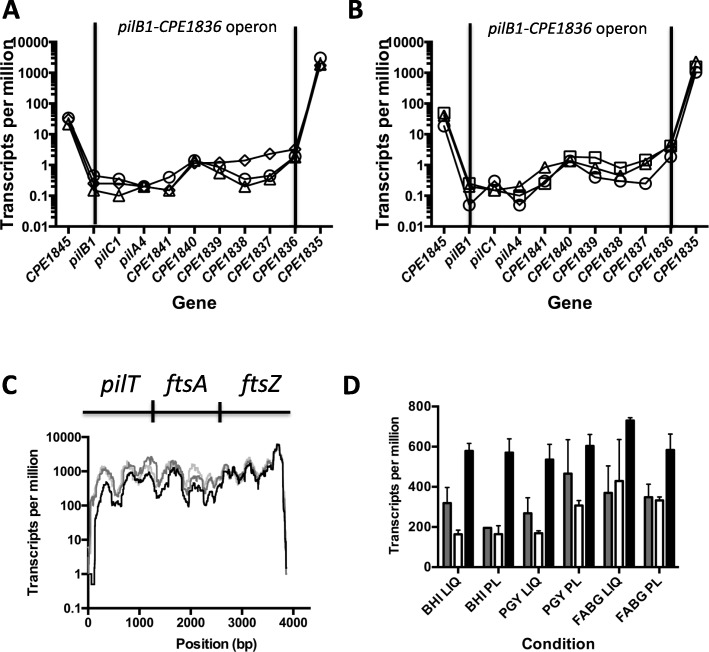
**a** and **b)** Transcript levels of genes in the *pilB1*- the gene encoding CPE1836 operon in cells grown on plates (**a**) and in liquid (**b**). Circles, BHI; triangles, PGY; squares, FABG. The vertical bars represent the boundaries of the proposed operon. Note the higher levels of transcripts seen in the flanking genes. Data points were connected by lines to illustrate trends in changes of transcript levels. **c** Base by base transcript levels of the *pilT*-*ftsA*-*ftsZ* operon from cells grown on plates. Black line, BHI; dark gray line, PGY, light gray line, FABG. A similar pattern was observed for cells grown in each medium in liquid. **d** Transcript levels for each gene in the *pilT*-*ftsA*-*ftsZ* operon under the conditions indicated on the X-axis. Gray, *pilT;* white, *ftsA;* black, *ftsZ*. PL, plates; LI, liquid. The mean and SEM are shown

The pilin operon beginning with *pilB1* (Fig. 1) was originally annotated as being composed of the four genes *pilB1*-*pilC1*-*pilA4*-the gene encoding CPE1841 [3, 9]. Analysis of the RNA-Seq data shows those four genes having very low levels of expression in both liquid (Fig. 7a) and plate (Fig. 7b) grown cells. The next five genes on the chromosome are transcribed in the same direction as the *pilB1* operon and also have the same low level of transcription under liquid and plate culture conditions (Fig. 7a and Fig. 7b). The operon prediction program, Database of Prokaryotic Operons (DOOR^2^, available at http://csbl.bmb.uga.edu/DOOR/index.php), predicts that in strain 13, these nine genes are composed of two separate operons, *pilB1*-*pilC1*-*pilA4*-the genes encoding CPE1841 as well as CPE1840-CPE1836 (Additional file 3: Figure S4A). However, in *C. perfringens* strain SM101, the DOOR^2^ software predicts all nine genes are in a single operon (Additional file 3: Figure S4B), and the same is true for each of the other sequenced strains of *C. perfringens* (data not shown). Given that there is no identifiable terminator after the gene encoding CPE1841, we hypothesize that in strain 13 all nine genes are in a single operon similar to the other strains.

### The *pilT*-*ftsA*-*ftsZ* genes comprise a coordinately regulated operon

Using RT-PCR methods, we had noted in a previous report that the *pilT* gene was co-transcribed with the *ftsA* gene in strain SM101 [20]. To determine if *pilT* was co-transcribed with *ftsA* and *ftsZ* in strain HN13, we measured the transcript levels at each base of the potential *pilT-ftsA-ftsZ* operon (Fig. 7c). Since the transcript levels were similar across the three different conditions for plate-grown cells (Fig. 7c) as well as liquid-grown cells (data not shown), we concluded that the *pilT* gene is co-transcribed with the *ftsA* and *ftsZ* genes under each of the conditions that we tested. This gene synteny is conserved in all of the *C. perfringens* strains that have been sequenced, as well as species of *Clostridium* that are phylogenetically related to *C. perfringens* (Additional file 3: Figure S5). Of the three genes in the operon, *ftsZ* had the highest levels of transcripts followed by *pilT* and then *ftsA* (Fig. 7d).

### Confirmation of promoters upstream of the *pilA2* and *pilB2* genes

**Fig. 8:**
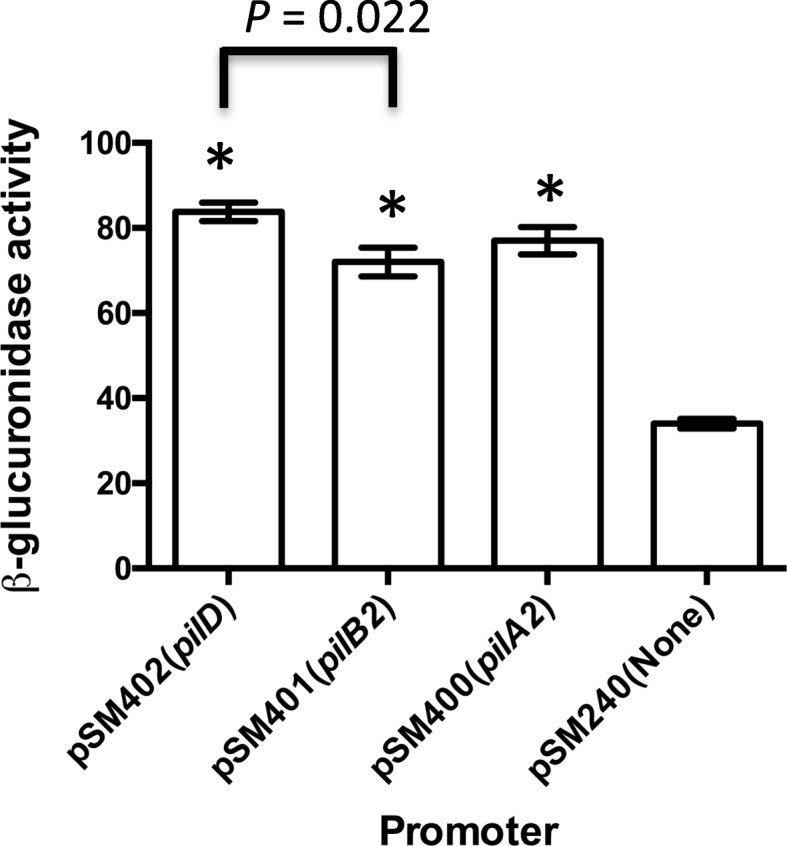
β-glucuronidase activity derived from pilin gene promoters. The values shown represent the mean and SEM of five independent samples of cells grown on PGY plates and processed as described in the Methods section. Asterisks indicate these vaules were significantly different (*P* < 0.001) from the pSM240 vector control using the two-tailed students t-test. The statistical difference between the measurements obtained from pSM402 and pSM401 (bar) were calculated using the two-tailed students t-test

We detected increased numbers of transcripts beginning upstream of the *pilA2* gene (Fig. 4 and Fig. 6a) and the *pilB2* gene (Fig. 4 and Fig. 5c) and identified putative promoters that could be responsible for this transcription (Fig. 6b and Fig. 5b, respectively). However, it is possible that this transcription is due to a promoter located upstream of the *pilD* gene and the different levels of transcripts for each gene is due to mRNA processing and degradation, as previously proposed ( [14, 16]. To answer these questions, we cloned the promoter regions of the *pilA2*, *pilB2*, and *pilD* genes upstream of a promoterless *gusA* gene into plasmid pSM240 [19]. pSM240 has four tandem terminators located upstream of putative promoters to block plasmid-originating transcription [19]. Bacterial colonies were grown on PGY plates and the edges scraped off to collect cells for β-glucuronidase assays, analogous to the methods used to collect cells for RNA seq experiments. The three promoters showed levels of β-glucuronidase activity 40–50 units above the empty vector control, with the *pilD* promoter slightly more active than the *pilB2* promote (Fig. 8). These assays confirm the existence of *pilA2*- and *pilB2*-specific promoters and suggest they are nearly equivalent in strength to that seen with the *pilD* promoter.

### Translational fusions of a reporter gene linked to the seven TFP-associated promoters indicates greater variation between media occurs on plates than in liquid

**Fig. 9:**
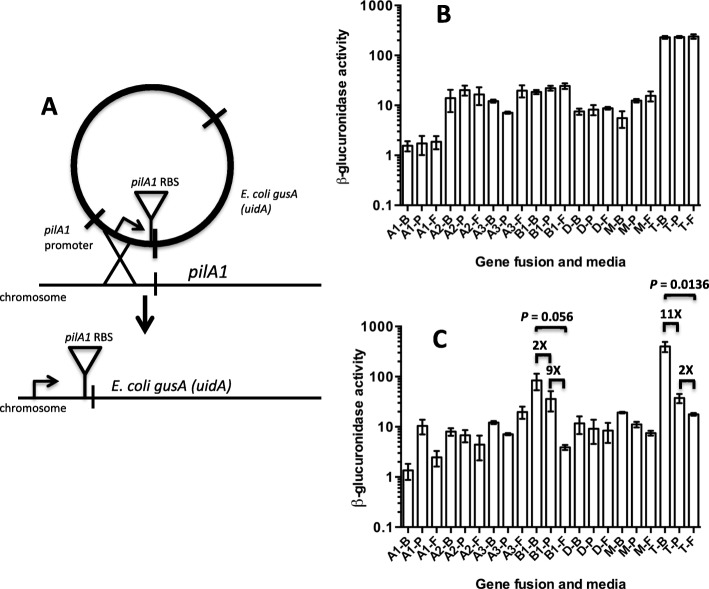
**a** Diagram illustrating the method for constructing translational promoter fusions to the *gusA* gene of *E. coli*. The *pilA1* promoter is shown as an example. **b** and **c** β-glucuronidase activity for each promoter-*gusA* fusion under the indicated conditions in liquid-grown cells (**b**) and plate grown cells (**c**). The media used were, B, BHI; P, PGY; F, FABG. Promoter fusion were to *pilA1* (A1), *pilA2* (A2), *pilA3* (A3), *pilB1* (B1), *pilD* (D), *pilM* (M), *pilT* (T). The mean and SEM of at least three independent samples are shown

To determine if translation of TFP-associated genes was proportional to the transcription levels of the corresponding genes, we designed constructs that could be integrated into the chromosome to report translational activity for each of the seven putative TFP promoters. The constructs were designed to retain the individual ribosomal binding site associated with the first gene after each promoter by coupling them to the *gusA* reporter gene (Fig. 9a). The bacteria containing these constructs were then grown in conditions identical to that used for the RNA-Seq experiments; that is, on BHI, PGY, and FABG plates and liquid. The cells grown in liquid varied only slightly in levels of transcription plus translation between the three different types of media for these promoters (Fig. 9b). However, cells grown on plates exhibited a much higher variation between conditions than those grown in liquid (Fig. 9c). In particular, the *pilB1* and *pilT* promoters exhibited 18- and 22- fold lower levels of β-glucuronidase activity, respectively, when comparing BHI and FABG plates, although the *pilB1* promoter differences were not quite significant (Fig. 9c).

### Plots of transcript levels vs β-glucuronidase activity show significant levels of post-transcriptional regulation on plates but not in liquid

**Fig. 10:**
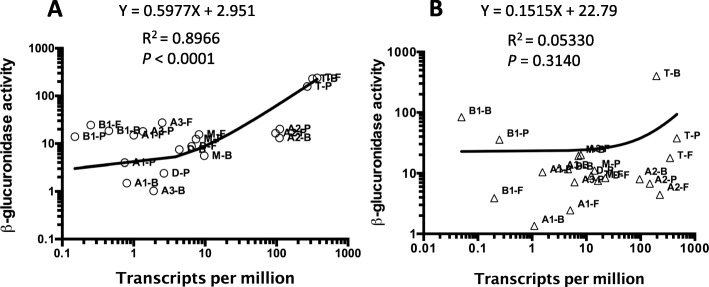
**a** and **b** Linear regression analysis of plots of TPM versus β-glucuronidase activity for each promoter-*gusA* fusion under the indicated conditions in liquid-grown cells (**a**) and plate grown cells (**b**). The media used were, B, BHI; P, PGY; F, FABG. Promoter fusion were to *pilA1* (A1), *pilA2* (A2), *pilA3* (A3), *pilB1* (B1), *pilD* (D), pilM (M), *pilT* (T). The line formulas, R^2^ and *P* values are shown for each data set. Note both panels are in log scales on each axis. The *P* values were calculated to determine if the slope is significantly non-zero

In principle, in the absence of any post-transcriptional effects, there should be a linear relationship between transcript levels and translation of the *gusA* (*uidA*) fusion constructs. To determine if this was the case, we constructed plots of TPM versus the β-glucuronidase activity of each of the seven promoters and the first gene downstream in cells grown in liquid and on plates for the three different types of media (Fig. 10). For liquid grown cells, there was a strong linear relationship between the number of transcripts from each gene and the β-glucuronidase activity for the corresponding medium, evident in the R^2^ value of 0.8966 as well as a y-intercept value of 2.951 (Fig. 10a). However, when the cells were grown on plates, the linear relationship between transcripts and β-glucuronidase activity was lost, as shown by an R^2^ value of 0.0533 and a y-intercept value of 22.79. This Y intercept value suggests that significant β-glucuronidase activity was measured at low transcripts levels for at least some of the promoters. This can be seen most dramatically for the *pilB1* promoter grown on BHI plates where the TPM were under 0.1 while the β-glucuronidase activity was close to 100 units (Fig. 10b). Altogether, it appears that gene expression in plate-grown cells is subjected to post-transcriptional regulation that is absent in cells grown in liquid.

To identify which of the promoters were exhibiting post-transcriptional regulation we plotted the TPM versus β-glucuronidase activity for each individual promoter (Additional file 3: Figure S6). With the exception of *pilM*, all of the promoters exhibited significant differences in slope and expression pattern between plate grown and liquid grown cultures. Although linear correlation calculations with just three points are not statistically robust, there was a negative correlation (i.e., negative slope) between TPM and β-glucuronidase activity in six of the seven promoters in plate-grown cells, the exception being the *pilA3* promoter (Additional file 3: Figure S6). The largest amount of discontinuity between the levels of transcription and translation was seen with the *pilB1* promoter, in which transcript levels were consistently very low but translation of the *gusA* gene was frequently high, especially for BHI (Fig. 10). When the β-glucuronidase activity was plotted against TPM levels for each of the seven promoters in the same media conditions, growth in liquid showed a linear relationship of transcription to translation, while only one (BHI) did so on plates (Additional file 3: Figure S7).

### The *pilT*-*gusA* fusion is regulated by temperature

**Fig. 11:**
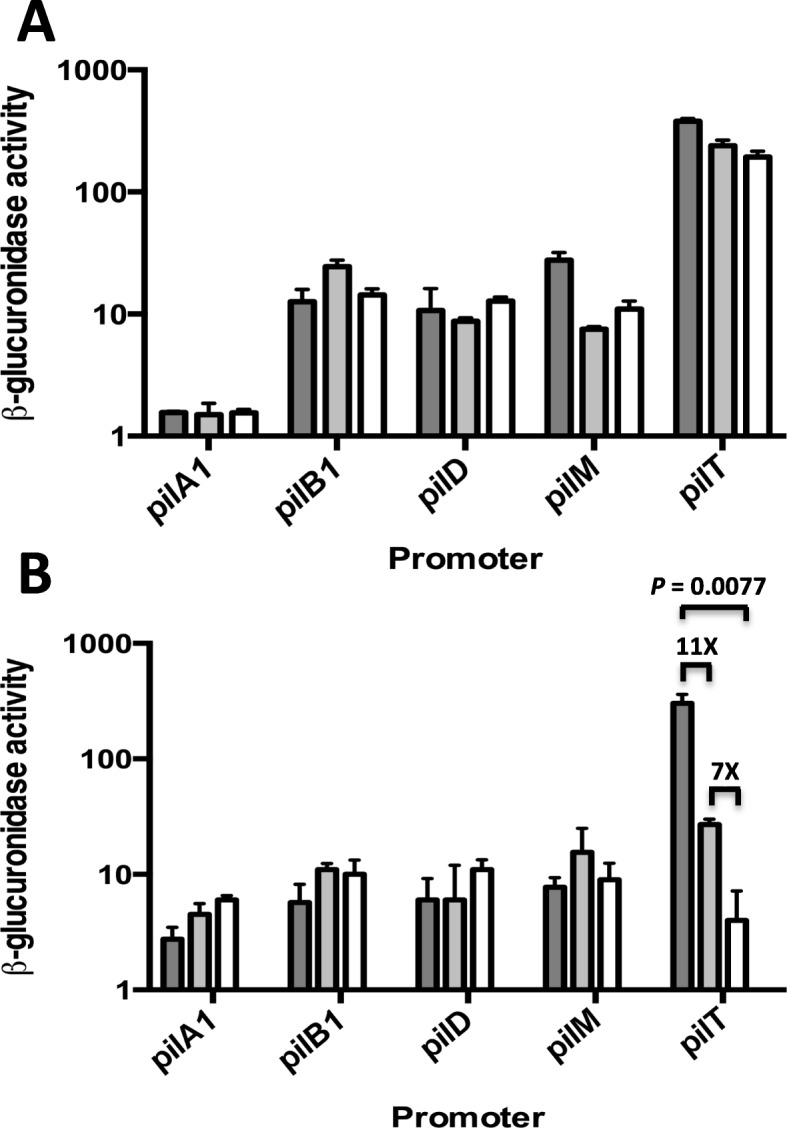
**a** and **b** β-glucuronidase activity for the promoter-*gusA* fusions indicated grown in liquid FABG (**a**) or FABG plates (**b**). 25 °C, dark gray bars; 37 °C, light gray bars; 45 °C, white bars. The mean and SEM of three independent experiments are shown

Since *C. perfringens* lives in a variety of different environmental conditions including soils, sediments, and in the intestines of birds and mammals [21], it was of interest to determine if TFP-associated gene expression was affected by growth temperature. To do this, we measured the β-glucuronidase activity from the *pilA1*, *pilB1*, *pilD*, *pilM*, and *pilT* promoters grown on FABG plates and liquid at 25 °C, 37 °C, and 45 °C. In liquid-grown cells, there was some variation in the *pilT* promoter in which activity declined two-fold with increasing temperature (Fig. 11a). In contrast, there was a significant decrease from 302 to four units in β-glucuronidase activity from the *pilT* promoter when the cells were grown on plates and the temperature was increased from 25 to 45 °C (Fig. 11b).

### The SigV sigma factor is involved in cell wall-dependent functions

We noted that locus *CPE0560*, which encodes a putative anti-SigV membrane bound protein, was expressed at higher levels on plates (Table 1). The gene encoding CPE0560 is the second gene in a likely two-gene operon with the *sigV* gene since the genes overlap and transcription is probably linked [11]. Anti-SigV proteins are membrane bound with a cytoplasmic domain that binds SigV in an inactive state [12, 22–24]. When the anti-SigV is degraded after the cell receives a specific environmental signal, SigV is released and can bind to RNA polymerase and initiate transcription at SigV-dependent promoters. SigV sigma factors are often involved in cell wall-associated functions. In *Clostridioides* (*Clostridium*) *difficile*, SigV controls the Dlt pathway, which is involved in D-alanylation of teichoic acids in response to the presence of lysozyme in the environment [24]. SigV is also linked to lysozyme resistance in *Bacillus subtilis* [25] and *Enterococcus faecalis* [26]. SigV functions have not been characterized in *C. perfringens.* To determine if SigV was needed for cell wall related functions in *C. perfringens*, separate deletions of the genes *sigV* and the gene encoding CPE0560 were constructed and the mutants examined for morphological changes. The *sigV* mutant cells were longer than the WT strain on all three types of plates, but the strain with a deletion in the gene encoding CPE0560 was the same length as the WT (Additional file 3: Figure S8). On PGY plates only, the *sigV* mutant produced numerous mini-cells at the terminus of the rod-shaped cells, suggesting a problem with cell division functions (Additional file 3: Figure S9 A-B). On FABG plates only, the *sigV* mutant made extended curved cells (Additional file 3: Figure S9 C-D), which contributed to the increase in average length seen on FABG plates (Additional file 3: Figure S8). However, no increase in sensitivity to lysozyme was observed with the deletion strains in *sigV* or the gene encoding CPE0560 (data not shown), suggesting the *C. perfringens* SigV protein may not play a role in lysozyme sensitivity as it does in *B. subtilis* or *E. faecalis*.

## Discussion

Surface-dependent phenotypes have not been studied to any extent in the Clostridia and other Gram-positive anaerobic bacteria. We explored this subject in *C. perfringens* because we had detected specific surface-associated phenotypes, primarily related to TFP functions. Here, we demonstrated that PilA2 was the primary pilin needed for adherence to murine myoblasts and that the ability to adhere was lost within minutes when bacteria were scraped off plates and suspended in liquid media (Fig. 2). We hypothesize that these two findings are linked. This rapid response suggests an environmental signal is somehow transmitted to the TFP assembly apparatus to change the levels of pilus polymerization. In a recent report, we demonstrated that purified assembly ATPase PilB2 from *C. perfringens* bound the second messenger molecule c-di-GMP and that increased levels of intracellular c-di-GMP led to increased levels of PilA2 on the surface of cells in a PilB2-and PilC2-dependent manner [27]. Diguanylate cyclases (DGC) synthesize c-di-GMP, which is then hydrolyzed by phosphodiesterases (PDE) [28]. *C. perfringens* strain 13 has a total of eight genes that encode DGCs, PDE or both [27]. We hypothesize that one of these DGC/PDEs acts as a sensor for the presence of a surface or another environmental clue encountered by bacteria on a surface and regulates PilB2 activity and pilus synthesis.

Western blots on membranes extracted from bacteria grown on BHI plates or liquid revealed a previously unreported change in the apparent molecular weight of the PilA2 protein, which we are interpreting as a type of post-translation modification (Fig. 2c). The modification occurred at higher levels in liquid-grown cells (Fig. 2d). Since liquid-grown cells showed reduced adherence, it is possible that this modification helps control the levels of PilA2 pilus polymerization in a negative fashion. Answering this question is beyond the scope of the current report but can be resolved once the nature of this modification is known and the genes involved identified.

We used RNA-Seq of cells grown on plates and in liquid for three different types of media to identify how the levels of transcription of TFP-associated genes varied in plate versus liquid-grown cells. The media were chosen because they varied in the nature and concentration of nutrients they contained. BHI is a low-glucose medium (0.3%), but glucose is in much higher concentrations (2%) in PGY and FABG. The FABG liquid culture contains 0.075% agar to increase the viscosity above that found in the other media. We anticipated that if a gene was expressed at higher levels (log_2_ > 2) in transcription on plates in all three media despite the large differences in medium components, it would be one that was responding to the cells being grown on a surface and not necessarily because of nutrient excess or limitation and, thus, may be part of a signal transduction pathway for sensing surfaces. There were hundreds of genes that were expressed at higher levels on plates versus liquid for each type of medium, but only 132 genes were expressed at higher levels on plates in all 3 media (Table 1). Therefore, we were successful in narrowing down the list of potential candidate genes that, in response to the growth on a surface, has its transcript level increased. Mutations introduced into one regulatory system that we found to be expressed at higher levels on plates, the SigV/anti-SigV complex, did indeed show significant morphological changes consistent with surface-dependent phenotypes we observed, such as increased cell length. Whether SigV directly regulates these phenotypes is unknown but the current evidence supports the hypothesis that it functions in a regulatory pathway that is activated on surfaces.

Genes encoding TFP-associated proteins in *C. perfringens* strain 13, the focus of this study, are found in three separate chromosomal loci (Fig. 1). For the main TFP locus, extending from *pilA1* to the gene encoding CPE2277, in all six conditions tested, the level of total transcripts varied but the relative proportions between the genes remained the same (Fig. 4). The level of transcription correlates with the proportion of proteins that one would expect to be present in a TFP apparatus. In this scenario, one would predict the proteins that comprise a Type IV pilus assembly apparatus would follow this general trend in protein stoichiometries: pilin (PilA2) > assembly ATPase (PilB2) > inner membrane core protein (PilC2) > inner membrane accessory proteins (PilM-PilN-PilO) [3]. In fact, the relative transcript levels do follow this pattern (Fig. 4). This strategy appears to be an efficient use of cellular resources, since mRNA is not made in excess of the amount of protein it encodes. How are the relative proportions of transcripts in the large TFP locus maintained? Our results indicate it is by the concerted action of promoters upstream of the *pilA1*, *pilD*, *pilB2*, *pilA2*, *pilM*, and *pilA3* genes acting in concert with terminators downstream of the *pilA1*, *pilA2* and the gene encoding CPE2277, as well as an intragenic terminator in the *pilC2* gene (Fig. 1).

Results from two other reports using Northern blots with *pilA2* gene probes suggested that the *pilA2* gene was in an operon with *pilD*-*pilB2*-*pilC2* with a promoter upstream of *pilD* [14, 16]. The authors hypothesized that the higher levels of *pilA2* transcripts were due to RNase Y processing of the *pilD-pilB2-pilC2-pilA2* transcript between the *pilC2* and *pilA2* gene, which led to stabilization of the *pilA2* transcript but degradation of the *pilD-pilB2-pilC2* mRNA [14, 16]. These authors also reported, as unpublished results, that they could not detect transcription or the presence of the PilA2 protein from a DNA fragment containing the *pilA2* gene and 200 bp upstream [14]. However, transcriptional fusions of the *pilD*, *pilB2*, and *pilA2* promoters to the *gusA* gene in a plasmid indicated the promoters were approximately equal in strength (Fig. 7). In addition, the chromosomal promoter fusions to the *gusA* gene showed similar levels of activity between the *pilD* and *pilA2* genes in cells grown on PGY plates (Fig. 9c), suggesting the plasmid-based promoters were acting in a similar fashion as those on the chromosome. Our results, in which an intragenic terminator in the middle of the *pilC2* gene lowered the expression 4-fold, suggests that under the conditions we tested, a promoter in the *pilC2*-*pilA2* intergenic region was responsible for increased *pilA2* transcript levels (Fig. 6a and b). The differences between the previous reports and this one may be due to the different methods used to detect promoters, RNA seq and *gusA* fusions here and Northern blots in [14, 16]. Overall, given the similar promoter activities, the transcript levels for the *pilD*, *pilB2*, and *pilA2* likely represent differences in the levels of mRNA degradation between the three genes, as previously proposed [14, 16].

We presented evidence that the operon beginning with *pilB1* likely contains nine genes and not the four genes originally annotated in it (Fig. 7a and b, Additional file 3: Figure S4). The function of this operon is still unknown, but it does contain an assembly ATPase (PilB1) along with an inner membrane core protein (PilC1) and at least one pilin, PilA4 (Fig. 1b). Therefore, it could assemble a pilus if it coordinates its activity with the accessory proteins PilM, PilN, and PilO, whose genes are located in the main pilus locus (Fig. 1a). An in-frame deletion of the *pilA4* gene did lead to a modest decrease in adherence to C2C12 myoblasts (Fig. 2), but the mechanism for this is unknown. None of the other six proteins encoded by the operon have identifiable functions, although two of them, CPE1841 and CPE1839, were identified by the PilFind program as having the characteristic N-terminal α-helix found in Type IV pilins, even though they lack sequence or structural prediction homology to other pilins [3].

Based on transcript levels across the three genes, the *pilT*-*ftsA*-*ftsZ* genes comprise a coordinately regulated operon. Why *C. perfringens* has evolved to have a TFP retraction ATPase in an operon with the essential cell division genes *ftsA* and *ftsZ* is unknown, but this implies there is some type of link between TFP functions and the divisome in *C. perfringens*. This synteny is conserved in related *Clostridium* species (Additional file 3: Figure S5) but not in more distant relatives such as *C. difficile*, where the *pilT* gene is located within the large TFP locus [3]. Species phylogenetically close to *C. perfringens*, such as *Clostridium novyi* and *Clostridium tetani*, have an additional gene between the *pilT* and *ftsA* genes (Additional file 3: Figure S5). This gene encodes a glycosyltransferase that has a potential function in cell wall biogenesis, which may be related to its location in an operon with *ftsA* and *ftsZ*. *C. perfringens* encodes an ortholog of this glycosyltransferase, CPE2071, but it is located outside of the *pilT* locus. The transcript levels of the three genes in the *pilT*-*ftsA*-*ftsZ* operon are not equal however, with *ftsZ* having the highest levels (Fig. 7d). This may correlate with the relative amounts of these proteins, since FtsZ has been shown to be in higher stoichiometries (4–5 fold) than FtsA in *E. coli* [29].

The transcription and translation of the *pilT* gene is complex. The TPM for *pilT* were relatively constant across the three media and from liquid to plate (Fig. 7d). Measurements of the *pilT* promoter using the *gusA* fusions told a different story, where there was no difference between media in liquid but a large difference on plates, with growth on BHI 11-fold higher than growth on PGY and 22-fold higher than growth on FABG (Fig. 9b and c). This is unlikely to be due to the presence of the *gusA* gene in place of the *pilT* gene, since the levels of expression were constant in liquid across the three media. It appears instead that translation was affected only on plates, and there was an inverse amount of expression with increasing richness (as measured by glucose levels) of the media. A similar pattern was seen in the regulation of the *pilT* transcription/translation with increasing temperatures, where there was only a modest decrease in expression from 25 °C to 45 °C in liquid, but this increased to 77-fold when the cells were grown on plates (Fig. 11). These results suggest that translation of *pilT*, and perhaps *ftsA* and *ftsZ*, is under stringent growth rate control in plate-grown cells.

Three reports have been published recently describing global RNA-Seq results on *C. perfringens* under different experimental conditions, and some of the results relate to TFP gene expression. RNA was extracted from planktonic and biofilm-grown cells from a chicken necrotic enteritis strain (CP4), and it was found that transcription of the *pilB2*, *pilC2*, and *pilM* genes were significantly reduced in the biofilm cells, suggesting that downregulation of transcription of the major TFP locus (Fig. 1) occurred in this strain [30]. RNA-Seq experiments on liquid-grown strain JIR325, a derivative of strain 13 (the parent of the strain used in these studies, HN13 [31]), and mutants lacking the global transcriptional regulators RevR and VirR indicated the *pilA1* gene was repressed by VirR [32]. However, the significance of this result in unknown, since we have yet to identify a function for the PilA1 protein in TFP assembly. (Fig. 2 and [3, 9]). Measurements of transcript levels from strain JIR325 extracted from infected mice in a myonecrosis model in comparison to liquid-grown cells showed increased transcript levels of the *pilT* and *pilC1* genes [33], although the *pilC1* gene was expressed at very low levels, similar to what we observed in this study (Fig. 7a-b). Transcript levels from in vitro and in vivo grown cells indicated transcript levels were *pilA2* > *pilB2* > *pilC2* (GEO repository files in GSE96890, referenced in [33]), which is identical to those described in this report (Fig. 4). These results suggest the main TFP locus is expressed at similar levels under in vitro and in vivo conditions, and the relative proportions of transcripts is held constant under a variety of conditions.

## Conclusions

This study reveals insights into how an anaerobic Gram-positive pathogenic bacterium responds to growth on surfaces, including the induction of transcriptional regulators and turning on multiple post-transcriptional regulatory mechanisms associated with TFP functions. The transcriptomics also revealed multiple metabolic adaptations to growth on surfaces in a congested environment, information that may be useful in devising strategies to prevent the spread of gas gangrene infections that occur in host tissues.

## Methods

### Bacterial strains and culture conditions

Bacterial strains, plasmids, and primers used in this study are listed in Additional file 1: Tables S1 and S2. *Escherichia coli* strain DH10B was grown in Luria Bertani broth at 37 °C for all transformations. When necessary, kanamycin and chloramphenicol were added to the media at a concentration of 100 μg/ml and 20 μg/ml, respectively. *C. perfringens* strain HN13, a Δ*galKT* derivative of strain 13 [31], was used as the wild type strain in this study. *C. perfringens* strains were grown anaerobically in PGY (30 g proteose peptone #3, 20 g glucose, 10 g yeast extract, 1 g sodium thioglycolate per liter), BHI (brain-heart infusion, Thermo Fisher), or FABG (LAB M fastidious anaerobe broth + 2% glucose) in an anaerobic chamber (Coy Laboratory Products, Inc.). Strain AH2 has an insertion of a suicide plasmid in the *bglR* gene of strain 13 encoding a β-glucuronidase and carries an erythromycin resistance gene [19]. Therefore, strains derived from AH2 which also contained the β-glucuronidase reporter gene vector pJV50 required 30 μg/ml erythromycin and 20 μg/ml chloramphenicol to maintain chromosomal insertions.

### Determination of bacterial cell length

Strain HN13 cells grown on BHI plates for 16 h and in liquid BHI to mid-log phase were isolated and placed on glass slides for microscopy. Phase contrast Images were collected using a climate-controlled Olympus IX71 inverted microscope equipped with a CoolSnap HQ2 CCD camera and DeltaVision deconvolution and image analysis software. The captured images were used to calculate the size of individual bacteria using the MicrobeTracker imaging suite [34] or ImageJ [35].

### Isolation of total RNA

*C. perfringens* HN13 cells were grown in duplicate independent experiments in six conditions: liquid culture to mid-log phase and 1% agar plates of PGY, BHI, and FABG media for 24 h. Liquid cultures were standardized to OD_600_ equal to 0.50. Cells were scraped from the outer edges of plate grown colonies and suspended in Dulbecco’s phosphate buffered saline (DPBS) to an OD_600_ of 0.50. Bacteria from both types of culture were pelleted and frozen in liquid nitrogen prior to RNA purification. Cells were lysed in 500 μl Tri Reagent (Zymo Research) using high-impact zirconium beads in a Mini-Beadbeater (Biospec) for 1 min and kept on ice. RNA was then purified from cell lysate using a Direct-Zol RNA Mini-Prep Plus Kit according to the manufacturer’s protocol (Zymo Research), including an on-column DNA digestion. RNA integrity was measured using an Agilent BioAnalyzer 2100 (Virginia Tech Biocomplexity Institute), and samples with a RIN of 8.3 or greater were used for RNA-Seq.

### RNA-Seq and gene expression analysis

Library construction was performed for Illumina sequencing by the Virginia Tech Biocomplexity Institute. All samples were processed with HiSeq Illumina sequencing creating 100-bp paired-end reads, and the resulting data was aligned to the *C. perfringens* strain 13 reference genome (NCBI, accession number NC_003366) using the bioinformatics read mapper Geneious version 9 with low sensitivity settings. For each sample, the total number of reads, total number of mapped reads, and the percentage mapped are listed in Additional file 1: Table S3. Relative expression levels of annotated coding regions were calculated by Geneious using units of transcripts per million (TPM). TPM, as proposed by Wagner [36], is proportional to the number of reads mapped to each coding sequence divided by the length of the coding sequence (the read coverage), normalized to the sum of the read coverages for all detected transcripts. Differentially expressed genes were identified by a stringent cutoff of log fold change of 2 or greater after filtering by a q value of 0.05 or less in TPM levels between different media conditions.

### qRT-PCR

A new set of RNA was extracted from wild type samples using the same growth and extraction conditions described above for RNA-Sequencing. RNA was quantified using a NanoPhotometer (Implen) and checked for quality using an Agilent BioAnalyzer 2100. All RIN values were above 8.2. The extracted RNA was converted to cDNA using a High Capacity cDNA Reverse Transcription kit (Life Technologies) per the manufacturer’s instructions. The cDNA was quantified using a NanoPhotometer (Implen), tested for purity by measuring absorbance ratios at 260/280 nm and 260/230 nm and used as the template in a 7300 Real-Time PCR System (Applied Biosystems/Life Technologies). Primer pairs (Additional file 1: Table S2) for five pilin genes of interest plus the control gene *lon* were designed using the software Primer Express, version 3 (Life Technologies) and optimized to 100% ± 10% efficiency using cloned coding regions of each gene as the template (Additional file 1: Table S2). Parameters for qRT-PCR primer design were as follows: 20–30 bp in length, 80–120 bp amplicon, 35% G + C content or higher, and T_m_ = 58 °C or higher (pairs not deviating by more than 1 °C). Template DNA (either plasmid or cDNA) was used at concentrations of 0.001 ng to 20 ng per 20 μl reaction containing 0.8 μl 10 μM specific forward and reverse primer, 10 μl 2x qPCRBIO SyGreen Mix Hi-ROX (PCRBiosystems), and 7.4 μl dH_2_O. Reactions were performed in MicroAmp Optical 96-well reaction plates in triplicate for each cDNA sample. Thermal cycler settings were programmed as follows: 95 °C for 2 min, 40 cycles at 95 °C for 5 s and 60 °C for 30 s, followed by a dissociation stage during primer optimization to confirm specific product amplification. Data was collected during stage 2 and analyzed through 7300 System SDS RQ software, version 1.4 (Life Technologies), using an automated cycle threshold, and relative expression level ranges were calculated using the ΔΔC_t_ method as described by the manufacturer (Applied Biosystems).

### Construction of in-frame gene deletions

In-frame deletions of the *pilA1*, *pilA2*, *pilA3* and *pilA4, sigV* and the gene encoding CPE0560 (anti-SigV) genes were made using the method of Nariya et al. [31], modified as described in Hendrick et al. [27]. The primers used to amplify the flanking DNA for each gene are listed in Additional file 1: Table S2. All deletions were confirmed by PCR across the deleted region.

### Construction of promoter-*gusA* fusions in the chromosome

The promoter regions of TFP operons and the reporter gene *gusA* (also called *uidA)* from *E. coli* were amplified and fused using overlapping PCR. The resulting PCR product and suicide vector pJV50 were digested using SalI-HF and PstI-HF, and the vector and PCR product were ligated using T4 DNA ligase (Promega). Transformants were screened for correct constructs using agarose gel electrophoresis, and a high concentration of plasmid DNA was extracted from the resulting strains using a ZymoPure Midi Prep Kit (Zymo Research) following the manufacturer’s protocol. Forty μg of suicide plasmid were electroporated into *C. perfringens* strain AH2 using the method described previously [19], and the chromosomal DNA from resulting strains was checked for homologous recombination via PCR.

### β-Glucuronidase assays

Reporter gene promoter fusion strains were utilized to assess promoter activity of TFP operons at their chromosomal loci. The β-glucuronidase assay was performed as previously described [37]. Briefly, cells were harvested from either the edges of colonies on plates or pelleted from a liquid culture in mid-log phase and suspended in 1 mL DPBS. OD_600_ was obtained, and cells were centrifuged for 5 min to pellet cells before suspending them in 0.8 mL buffer (50 mM NaHPO_4_ [pH 7.0], 1 mM EDTA, 5 mM dithiothreitol). Eight μl of toluene were added to the cells, which were vortexed for 1 min and put on ice for 10 min. Samples were then placed in a 37 °C water bath with caps open for 30 min. The assay was initiated by addition of 160 μl 6 mM 4-nitrophenyl D-β-glucuronide (Sigma Chemical Co.), and after further incubation, the reaction was halted by addition of 400 μl 1 M Na_2_CO_3_. Cellular debris was pelleted for 10 min, and A_405_ was measured in a Genesys 10S UV-VIS spectrophotometer (Thermo Scientific). The specific activity of β-glucuronidase enzyme in each sample was calculated using the following equation: specific activity = (A_405_ × 1000)/(OD_600_ x time [minutes] x culture volume [milliliters]).

### Construction of promoter transcriptional fusions to the *gusA* gene in the promoter less vector, pSM240

The putative promoter-containing regions upstream of the *pilA2*, *pilB2*, and *pilD* genes were amplified by PCR using the primers listed in Additional file 1: Table S2. The *pilA2*, *pilB2*, and *pilD* promoter regions were 226, 162, and 245 bp upstream of the ATG stop codon, respectively, and contained KpnI and PstI restriction sites. The PCR products and plasmid pSM240 [19] were digested with KpnI and PstI and ligated to form plasmids pSM400 (*pilA2*), pSM401 (*pilB2*), and pSM402 (*pilD*). Each of these plasmids were transformed into strain AH1, a derivative of strain 13 in which the endogenous β-glucuronidase-encoding gene (*bglR*) was mutated [19]. To measure promoter activity, the cells were grown on PGY plates overnight, cells were scraped from the colony edge and β-glucuronidase assays performed as described above.

### Construction of a PilC2-His_6_ expression vector

The *pilC2* gene from strain 13 was amplified with primers OAH117 and OAH118, which added PstI and SalI sites to the 5′ and 3′ end of the gene, along with an additional 6 His-encoding codons at the 3′ end of the gene (Additional file 1: Table S2, Supplemental Material Tables and Text). The PCR product was ligated to the PCR cloning vector pGEM-T Easy, digested with PstI and SalI and ligated to PstI-SalI digested pKRAH1, a vector used for lactose-inducible expression [19].

### Myoblast adherence assays

Adherence of *C. perfringens* strains to C2C12 cells took place in a Coy anaerobic chamber with an atmosphere of 85% N_2_, 10% CO_2_, and 5% H_2_. C2C12 cells were grown to confluency (~ 2 days) in 0.5 ml DMEM/FBS in 48-well tissue culture plates and then placed in a 37 °C incubator inside the anaerobic chamber. *C. perfringens* strains, grown overnight on BHI medium with agar [37] under anaerobic conditions, were removed from the anaerobic chamber, scraped off the plates and suspended in 1 ml DPBS. Bacteria were pelleted in a centrifuge, suspended in DPBS, and the suspensions were diluted in DPBS to give ~ 2 × 10^7^ cfu /ml. The number of cfu in the suspension was determined by serial dilution and plating on BHI plates. After the C2C12 cells were in anaerobic conditions for 2 h, five μl of the bacterial suspension (~ 1 × 10^5^ cfu) was added to each well and incubated anaerobically at 37 °C for 75 min. The plates were then removed from the anaerobic chamber, and each well was washed three times with 0.5 ml aerobic DPBS to remove unattached bacteria. After the final wash, 0.5 ml of distilled water was added to the wells to lyse the myoblasts. The cells and bacteria were scraped off the bottom of the well, placed in a microcentrifuge tube, and subjected to vortex mixing for 20 s. The bacteria in the sample were then quantified by serial dilution and plating on BHI medium. Attachment assays for each strain were performed on quintuplicate samples from at least three separate experiments. For the experiment in which cells were grown on plates but then suspended in liquid before measuring adherence, the cells were scraped off BHI plates after 16 h of anaerobic growth and suspended in tubes containing 2 ml of anaerobic BHI liquid medium. At the indicated times, the tube was removed from the chamber, the cells were pelleted by centrifugation and suspended in 1 ml DPBS. Five μl of this suspension was added to the C2C12 cells in the anaerobic chamber and adherence was measured as described above. The number of cfu in the suspension was determined by serial dilution and plating on BHI plates.

### Western blots

For anti-PilA2 Western blots, membranes were prepared from bacteria grown on BHI plates anaerobically at 37 °C for 16 h or from cells grown to mid-log in BHI liquid medium. For plates, cells were scraped the off and suspended in 0.5 ml resuspension buffer (100 mM Tris, pH 7.1). For liquid, 10 ml of cells were pelleted by centrifugation and suspended in 0.5 ml resuspension buffer. The cell suspensions were then placed in 2 ml centrifuge tubes containing 0.1 mm diameter zirconium beads (Benchmark Scientific) and shaken in a Beadbeater device (Biospec) for two one-minute cycles and placed on ice. The beads were removed by centrifugation at 2000 x g for 1 min and the supernatant was removed and centrifuged at 15,000 x g for 2 min to remove unbroken cells. The supernatant was removed and centrifuged 100,000 x g for 1 h to pellet membranes, and the resulting membranes were suspended in resuspension buffer. As previously described [38], the OD_600_ of each membrane suspension was measured in a spectrophotometer (Genesys 10S UV-VIS spectrophotometer, Thermo Scientific) to allow equivalent amounts of membranes to be used for SDS-PAGE. Membranes were heated for 15 min at 95 °C after the addition of 4 x SDS sample buffer (200 mM Tris-Cl [pH 6.8], 100 mM DTT, 8% SDS, 0.4% bromophenol blue, and 40% glycerol). Samples were then run on SDS-PAGE gels, and proteins were transferred from the gel onto the PVDF membrane according to the manufacturer’s (Bio-Rad Trans Blot-Turbo) instructions. The PVDF membranes were placed in a SNAP i. d. 2.0 protein detection apparatus (Millipore) then blocked with 2% BSA and 0.5% gelatin in Tris-buffered saline with Tween (TBST, Santa Cruz Biotechnology) for 10 min. Affinity-purified rabbit anti-PilA2 antibody was added at a 1:1000 dilution in TBST for 10 min, and the membranes were then washed 4 times with TBST. Goat-anti-rabbit-HRP conjugate antibody diluted 1:5000 in TBST was added for 10 min, followed by 4 washes with TBST. The chemiluminescence substrate SupersSignal West Dura Extended Duration Substrate (Thermo Scientific) was added to the membrane, and light emission was detected with a Chemi-Doc MP Imaging System (Bio-Rad). For PilC2 Western blots, the samples were processed in the same manner as described above for PilA2 (both proteins are membrane bound). The methods used for Western blotting were the same, except rabbit anti-PilC2 and mouse anti-His [6] antibodies (1:200) were used as the primary antibodies, and the secondary antibodies were goat-anti-rabbit Dylight 550 (1:5000) and goat-anti-mouse Starbright 700 (1:5000) (both from Bio-Rad), respectively. The production of rabbit polyclonal antibodies against PilC2 was previously described [27]. Affinity-purified rabbit polyclonal antibodies against PilA2 were made by Genscript using a peptide (N-CVFAVEVSGKEDSPV-C) specific for residues 110–123.

### Statistics

The sample number and statistical tests applied for each experiment are shown in the figure legends of the respective figures. All statistical calculations were carried out using GraphPad Prism 6 software.

## Supplementary information

**Additional file 1.** Tables S1-S4.

**Additional file 2.** Table S5.

**Additional file 3.** Figures S1-S9.

## Data Availability

All data generated or analyzed during this study are included in this published article and its supplementary materials. The data discussed in this publication have been deposited in NCBI’s Gene Expression Omnibus [39] and are accessible through GEO Series accession number GSE99224 (https://www.ncbi.nlm.nih.gov/geo/query/acc.cgi?acc=GSE99224).

## Abbreviations

BHI: Brain heart infusion
cfu: colony forming unit
FABG: Fastidious anaerobic broth plus glucose
FDR: False discovery rate
PGY: Proteose peptone-glucose-yeast extract
TFP: Type IV pili
TPM: Transcripts per million
WT: Wild type strain
