# Supplementary material for: Changes in the expression of genes encoding type IV pili-associated proteins are seen when Clostridium perfringens is grown in liquid or on surfaces
Source: BMC Genomics. 2020 Jan 14;21:45. doi: 10.1186/s12864-020-6453-z (PMC6958937; doi:10.1186/s12864-020-6453-z)
Supplement: Supplementary file 1 — Additional file 1. Tables S1-S4. [file 12864_2020_6453_MOESM1_ESM.pdf]

Supplemental Material

Table S1 Strains and plasmids used in this study.

Strain or plasmid (parent)	Relevant genotype	Selection/ Resistance	Source
Escherichia coli			
DH10B	F^- *mcrA*Δ *mrr-hsdRMS mcrBC* ϕ80d*lac*ZΔM15 *lacX74 deoR recA1 araD139Δ ara, leu7697 galU galK*Δ*rpsL endA1 nupG*	None	Gibco/BRL
Clostridium perfringens			
HN13	Wild type; *galKT*-	None	(1)
AH2	Mutant in *bglR*; *uidA* fusion parent strain	Erm	(2)
AH7	Δ*pilA1*, from HN13	None	This study
AH8	Δ*pilA2*, from HN13	None	This study
AH9	Δ*pilA3*, from HN13	None	This study
AH10	Δ*pilA4*, from HN13	None	This study
SRM4	*pilA1* operon promoter fused to *uidA* in HN13	Erm Cm	This study
SRM9	*pilA2* operon promoter fused to *uidA* in HN13	Erm Cm	This study
SRM10	*pilA3* operon promoter fused to *uidA* in HN13	Erm Cm	This study
SRM5	*pilB1* operon promoter fused to *uidA* in HN13	Erm Cm	This study
SRM6	*pilD* operon promoter fused to *uidA* in HN13	Erm Cm	This study
SRM7	*pilM* operon promoter fused to *uidA* in HN13	Erm Cm	This study
SRM8	*pilT* operon promoter fused to *uidA* in HN13	Erm Cm	This study
Plasmids			
pSRM18	*cpe1788* in pKRAH1	Cm	This study
pSRM8	*pilA1::uidA* in pJV50	Cm	This study
pSRM22	*pilA2::uidA* in pJV50	Cm	This study
pSRM23	*pilA3::uidA* in pJV50	Cm	This study
pSRM10	*pilB1::uidA* in pJV50	Cm	This study
pSRM12	*pilD::uidA in* pJV50	Cm	This study
pSRM14	*pilM::uidA in* pJV50	Cm	This study
pSRM16	*pilT::uidA in* pJV50	Cm	This study
pJV50	Suicide vector used for *uidA* fusion strains	Cm	(3)
pKRAH1	Lactose inducible expression vector	Cm	(2)
pAH10	*pilA2* in pKRAH1	Cm	Andrea Hartman
pKRAH1-*pilB2*	*pilB2* in pKRAH1	Cm	Andrea Hartman
pKRAH1-*pilC2*	*pilC2* in pKRAH1	Cm	Andrea Hartman
pKRAH1-*pilT*	*pilT* in pKRAH1	Cm	Andrea Hartman
pKRAH1-*cpe2277*	*cpe2277* in pKRAH1	Cm	Andrea Hartman
pSRM24	*lon* in pKRAH1	Cm	This study
pSRM35	Promoterless *sr79* in pKRAH1	Cm	This study
pSM240	Vector for measuring promoter activity in *C. perfringens*	Cm	(2)
pSM400	*pilA2* promoter region in pSM240	Cm	This study
pSM401	*pilB2* promoter region in pSM240	Cm	This study
pSM402	*pilD* promoter region in pSM240	Cm	This study

Table S2 Primers used in this study.

Primer	Sequence (5' – 3')
pilA1::*uidA*	
OSRM24	gtaGTCGACttattttgtagacaactgggaaaaactgtg (SalI site)
OSRM25	cacgggttggggtttctacaggacgtaacatcagtaataacatttatggtcctcc
OSRM26	ggaggaccataaatgttattactgatgttacgtcctgtagaaaccccaacccgtg
pilA2::uidA	
OSRM50	cactagGTCGACtattagataaaacagcagatttttatgatgatgaac (SalI site)
OSRM51	ggaaaaccaatgaatacaatgttacgtcctgtag
OSRM52	ctacaggacgtaacattgtattcattggttttcc
pilA3::*uidA*	
OSRM53	ggaGTCGACactggaaatccagattctacttacttattagtg (SalI site)
OSRM54	ctacaggacgtaacataaattttcatttaatcacc
OSRM55	ggtgattaaatgaaaaatttatgttacgtcctgtag
pilB1::*uidA*	
OSRM27	gatGTCGACggaagctatactttagaaaagcaaatag (SalI site)
OSRM28	cacgggttggggtttctacaggacgtaacatatatttcatatatcaacctcctc
OSRM29	gaggaggttgatatatgaaatatatgttacgtcctgtacaaaccccaacccgtg
pilD::*uidA*	
OSRM30	ggatagGTCGACcgctaaggcaaaagtagataacaaaattg (SalI site)
OSRM31	cacgggttggggtttctacaggacgtaacattatagacacattgcctcccaaatcatcc
OSRM32	ggatgatttgggaggcaatgtgtctataatgttacgtcctgtagaaaccccaacccgtg
pilM::*uidA*	
OSRM33	catcaGTCGACgctttggttgctgaaggtaagataataggagg (SalI site)
OSRM34	cacgggttggggtttctacaggacgtaacattattgccaaaactttccctcctttacc
OSRM35	ggtaaaggagggaaagttttggcaataatgttacgtcctgtagaaaccccaacccgtg
pilT::*uidA*	
OSRM36	caaGTCGACgggaatacgacttgcaacatatgcagc (SalI site)
OSRM37	cacgggttggggtttctacaggacgtaacataxtttgcataaatctcctcctaaac
OSRM38	gtttaggaggagatttatgcaaagtatgttacgtcctgtagaaaccccaacccgtg
All *uidA* fusion C-termini	
OSRM39	gttCTGCAGgattcattgtttgcctccc (PstI site)
pilA2 qRT-PCR	
OSRM62	tcaaatgctaaagacgacgttacag
OSRM63	tctactgcaaatacaccaccatcaa
pilB2 qRT-PCR	
OSRM78	tgtgcgtccgtatatagctgt
OSRM79	gcttttgttaattcttccgctgc
pilC2 qRT-PCR	
OSRM76	tgaaaaagagaxaagataagaggaaaga
OSRM77	gcaataatagctaccactccaagaact
pilT qRT-PCR	
OSRM64	gaatgagaacgatggacatggaa
OSRM65	gcacaattcattgccgtttctt
cpe2277 qRT-PCR	
OSRM68	tctgtaggaagttggaagtggagtt
OSRM69	tcttacattgggtaaatctccaacac
lon qRT-PCR	
OSRM70	ggtagttggtcaagagagagctgtta
OSRM71	caacccctggaggtccataa
pSRM35	

OSRM108	GTCGACgaaaagtttatgaaaacaggttatttagaaattaattg (SalI site)
OSRM95	gctaaaacaacagttaattctattaatgtGGATCCccc (BamHI site)
Δ*pilA1*	
OAH129 (5' primer for upstream fragment)	gtcgacgtgatgcaggtgtaggatttgatgc
OAH130 (3' primer for upstream fragment)	gtatatttatactatagacaccgtaacttttcttcctgct ttcagtaataacatttatggtcctcc
OAH131(5' primer for downstream fragment)	ggaggaccataaatgttattactgaaagcaggaagaaaag ttacggtgtctatagtataaatataccc
OAH132 (3' primer for downstream fragment)	ggatccgctaatactgctattattccaccaataataaatg
Δ*pilA2*	
OAH133 (5' primer for upstream fragment)	gtcgaccaaacttacttactagcatgattgatg c
OAH134(3' primer for upstream fragment)	gattatttctttcattagttactgatgcgttttgtttttt tgtattcattggttttcc
OAH135 (5' primer for downstream fragment)	ggaaaaccaatgaatacaaaaaaacaaaacgcatcagtaa ctaatgaaagaaataatc
OAH136 (3' primer for downstream fragment)	ggatcccaccagattttgtagtttttataagtattaac
Δ*pilA3*	
OAH137(5' primer for upstream fragment)	gtcgaccaaaagatttaggcaatggatcaagtttag
OAH169 (3' primer for upstream fragment)	cacctccaacccatcttttatttttatttctatttttca tttaatcaccatctttctc
OAH170 (5' primer for downstream fragment)	gagaaagatggtgattaaatgaaaaatagaaataaaaaataaaagatgggttggaggtg
OAH140 (3' primer for downstream fragment)	ggatccggcaaatggttaacatcgtaagatctaaatg
Δ*pilA4*	gagaaagatggtgattaaatgaaaaatagaaataaaaaat aaaagatgggttggaggtg
OAH141(5' primer for upstream fragment)	gtcgacactgtaaaagataagaaatacaaagaatccc
OAH142 (3' primer for upstream fragment)	cctcataaatcacttaacattaattttccatatgcctctt ttactatacatatttcatgccctcaaagg
OAH143 (5' primer for downstream fragment)	cctttgagggcatgaaatatgtatagtaaaagaggcatat ggaaaattaatgttaagtgatttatgagg
OAH144 (3' primer for downstream fragment)	ggatccataacaataaatcctttcttcttgatttttaacc
Δ*sigV*	
OSRM84 (5' primer for upstream fragment)	ggtgtcgacccagaagaggttgctaaagc
OSRM85 (3' primer for upstream fragment)	gctaggaggggaaagatggaaaaaactatgggctatttaaatgaataaaaaagacttag
OSRM86 (5' primer for downstream fragment)	ctaagtcttttttattcatttaaatagcccatagtttttccatctttcccctcctagc
OSRM87 (3' primer for downstream fragment)	ccgtaaaacttactttagcttctgcagcatagggatcctgc
Δ*CPE0560*	
OSRM88 (5' primer for upstream fragment)	5' ggcGTCGACatatgttaaagatgagcaggatgctcttgaag 3' (SalI site)
OSRM89 (3' primer for upstream fragment)	5' ggagggctatttaaatgaataaaaaagacggaaataaatttagaataaacaaggtttat 3'
OSRM90 (5' primer for	5' ataaaccttgtttattctaaatttatttccgtcttttttattcatttaaatagccctcc 3'

downstream fragment)	
OSRM91 (3' primer for downstream fragment)	5’ ctcGGATCCgtagcacagtgagtaactgcagctacg 3’ (BamHI site)
pKRAH-pilC2	
OAH117	5’-CTGCAGtaattaggtgaaagaaaaggagaggaattatggc-3’
OAH118	5’-GTCGACttaatgatgatgatgatgatgacctatactgttatacattttaaacataggtg-3’
pilA2 promoter	
OSM354	5’-cagcaGTCGACtatgatgatgaactagaaagagaaatac -3’
OSM355	5’-ccCTGCAGtttttttgtttttttgtattcattggttttcc -3’
pilB2 promoter	
OSM356	5’- gGTCGACatatagcctttggaccagctatagg-3’
OSM357	5’-cCTGCAGcaattagtatatctcctaaacgctttttc -3’
pilD promoter	
OSM358	5’-gGTCGACtataaaatataataataaaacttattttactacatgccc -3’
OSM359	5’-ccCTGCAGaaaaaatattatagacacattgcctcccaaatc -3’

Table S3 Sample coverage.

Sample	Total Reads	Mapped Reads	Percentage of Mapped Reads
BHILI1	18,534,996	16,302,909	88%
BHILI3	23,566,774	21,879,774	93%
BHIPL1	22,280,318	18,994,017	85%
BHIPL2	20,387,754	18,145,757	89%
PGYLI1	22,390,086	18,006,491	80%
PGYLI2	20,745,536	18,771,451	90%
PGYPL1	23,451,252	17,847,747	76%
PGYPL2	20,099,696	18,178,362	90%
FABGLI1	19,798,656	17,448,863	88%
FABGLI2	23,316,700	21,358,386	92%
FABGPL1	24,439,042	20,171,471	83%
FABGPL2	21,927,608	20,383,062	93%

LI, liquid; PL, plate

Table S4 Numbers of genes with differential expression in various media in crosswise comparisons.

	BHI	PGY	FABG	FABG/PGY	PGY/ BHI	FABG /BHI	BHI/FABG	PGY/FABG	BHI/ PGY	Total
Plates	469	926	429	257	965	990	327	432	158	4953
Liquid	902	90	521	861	145	609	250	56	537	3971

Each comparison is based on two-fold or greater average transcripts per million (TPM) in each growth medium (BHI, PGY, or FABG) in either liquid-grown cells or plated cultures. For single comparisons, TPM was averaged for each gene for two samples, and differential expression was found by comparing plate and liquid values for each gene. For dual comparisons, the plate and liquid averages for every gene in the various media were compared.

References used in Supplemental Material

1. Nariya H, Miyata S, Suzuki M, Tamai E, Okabe A. Development and application of a method for counterselectable in-frame deletion in Clostridium perfringens. Applied and environmental microbiology. 2011;77(4):1375-82.
2. Hartman AH, Liu H, Melville SB. Construction and characterization of a lactose-inducible promoter system for controlled gene expression in Clostridium perfringens. Applied and environmental microbiology. 2011;77(2):471-8.
3. Therit B, Cheung JK, Rood JI, Melville SB. NanR, a Transcriptional Regulator That Binds to the Promoters of Genes Involved in Sialic Acid Metabolism in the Anaerobic Pathogen Clostridium perfringens. PloS one. 2015;10(7):e0133217.
