# Supplementary material for: Changes in the expression of genes encoding type IV pili-associated proteins are seen when Clostridium perfringens is grown in liquid or on surfaces
Source: BMC Genomics. 2020 Jan 14;21:45. doi: 10.1186/s12864-020-6453-z (PMC6958937; doi:10.1186/s12864-020-6453-z)
Supplement: Supplementary file 3 — Additional file 3. Figures S1-S9. [file 12864_2020_6453_MOESM3_ESM.pdf]

Figure S1

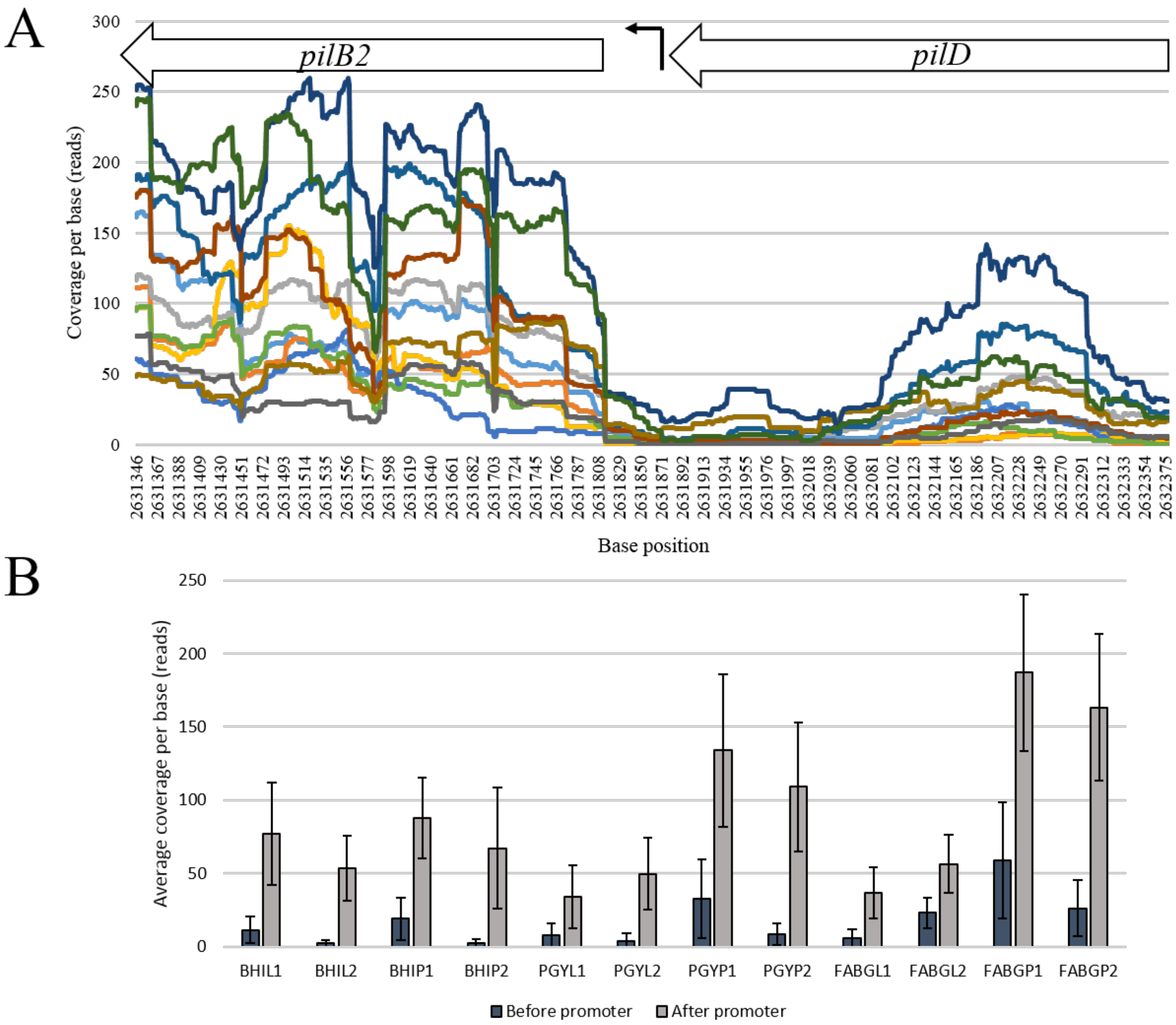

FIGURE S1. Promoter region located upstream of *pilB2*. (A) Coverage map of transcripts aligning 500 bp upstream and downstream of the suspected promoter between *pilD* and *pilB2* for all twelve samples. (B) Average number of transcripts aligning per base 500 bp upstream of *pilB2* promoter (before, blue) and 500 bp downstream of promoter (after, grey) for twelve samples (P, plate; L, liquid). Means and standard deviations are shown. All differences are significantly different using Student's t-test ($P < 0.001$).

Figure S2

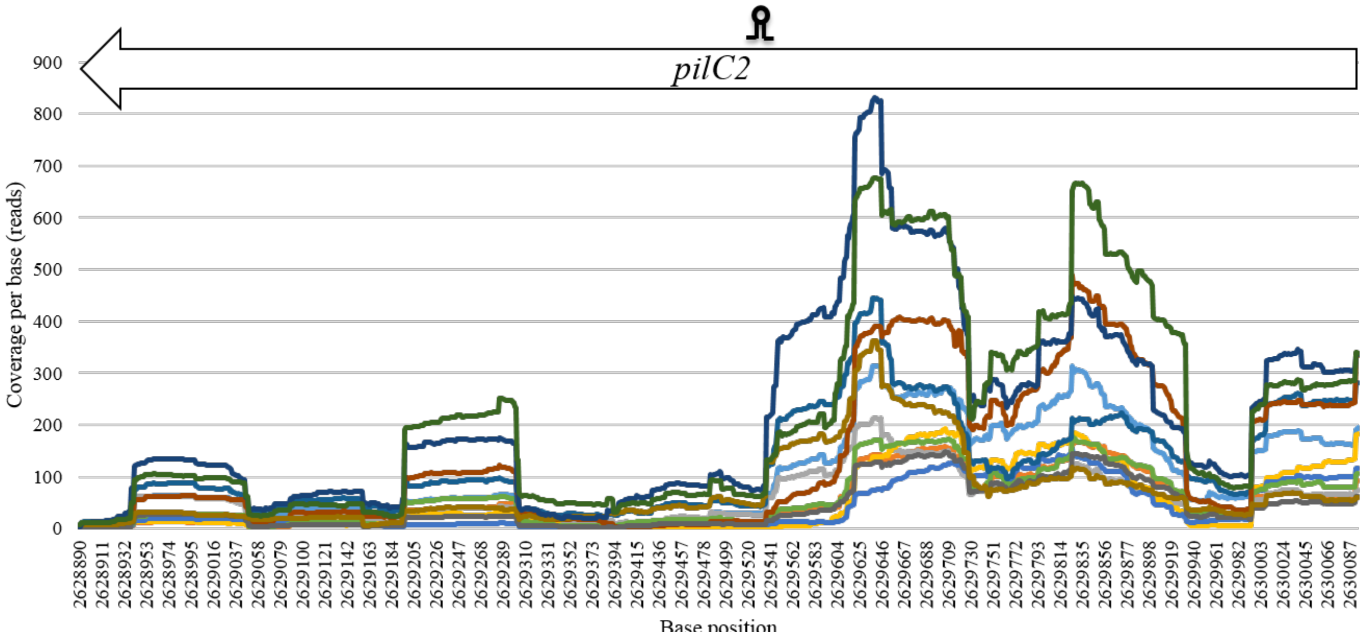

FIGURE S2. Termination during coding sequencing of *pilC2*. Coverage map of transcripts aligning upstream and downstream of the suspected terminator interrupting *pilC2* coding region for all twelve samples.

Figure S3

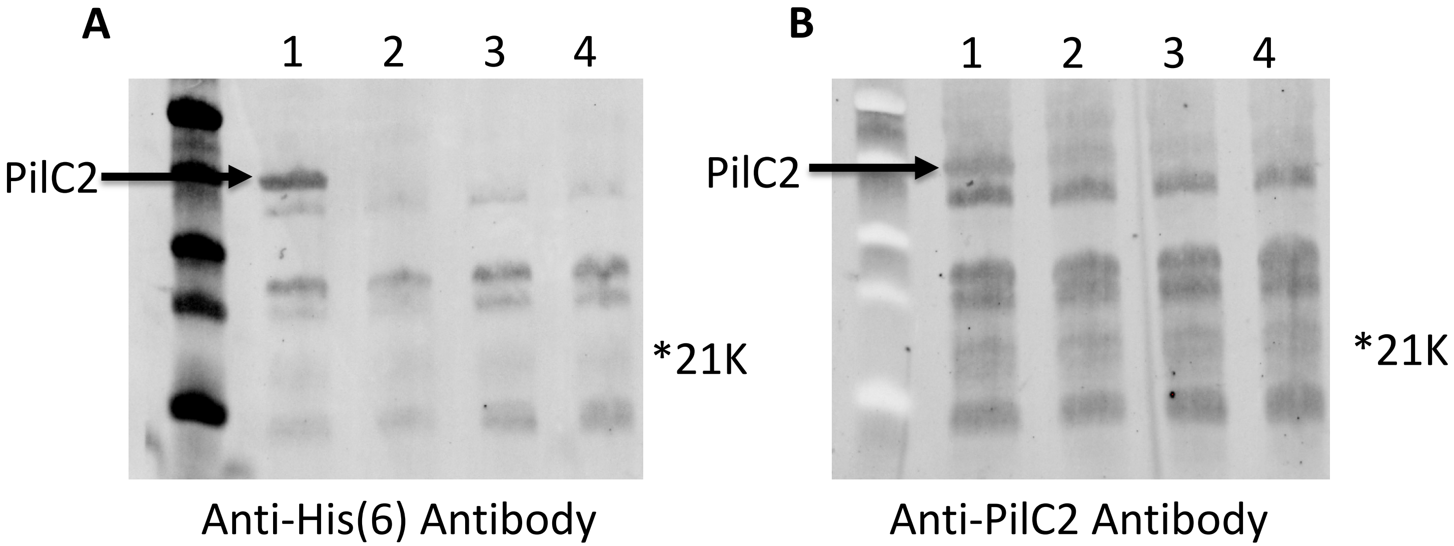

Lane 1: Strain 13 pKRAH-pilC2 (+ lactose)
Lane 2: Strain 13 pKRAH-pilC2 (- lactose)
Lane 3: Strain 13 (+ lactose)
Lane 4: Strain 13 (- lactose)

FIGURE S3. Western blots showing the presence of only the full length PilC2 proteins when the *pilC2-*$_{his6}$ gene was induced by the addition of lactose. Whole cell extracts were prepared from cultures with equivalent OD_{600} using the zirconium beads and Bead Beater device described in the Experimental Procedures. The asterisk denotes the predicted size of a truncated PilC2 protein but no bands were seen at this mol wt.

Figure S4

A

B

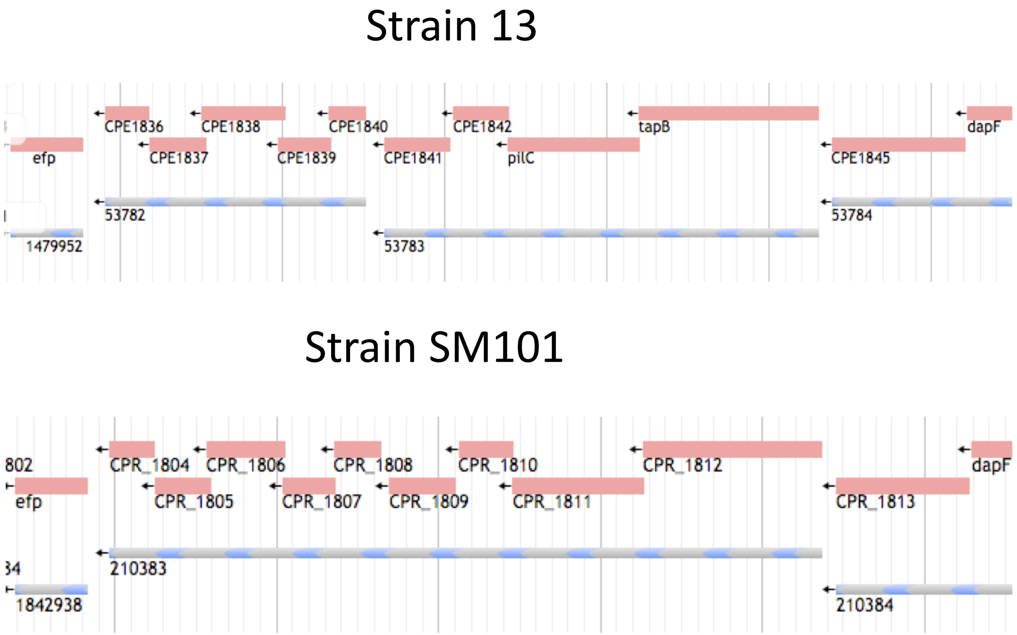

FIGURE S4. Screen capture of the *pilB1-CPE1836* operon prediction from the Database of Prokaryotic Operons (DOOR[2], available at http://csbl.bmb.uga.edu/DOOR/index.php) for strain 13 (panel A) and strain SM101 (panel B). The gene CPR_1812 corresponds to the tapB (pilB1 gene) and so on.

Figure S5

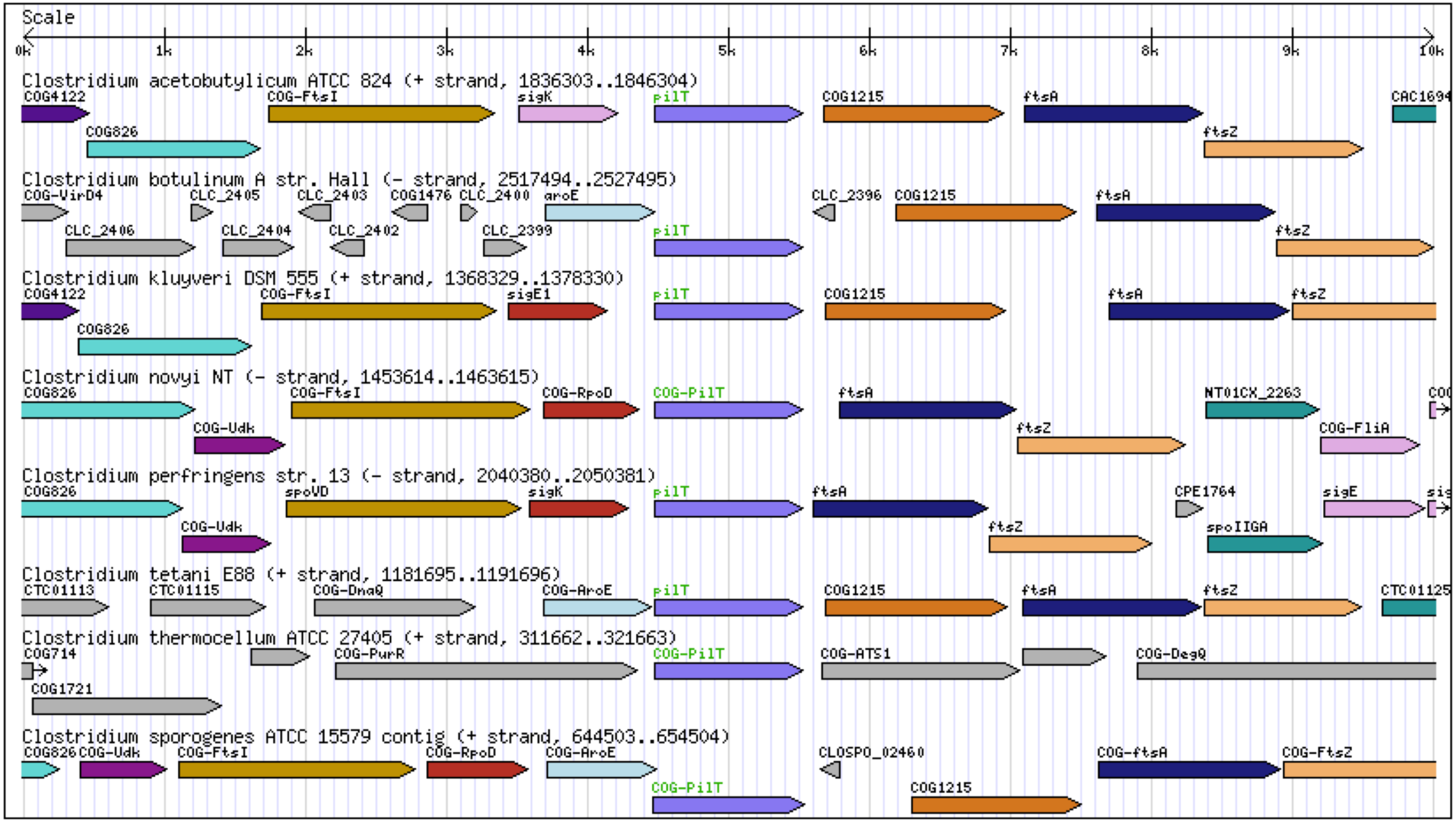

FIGURE S5. Synteny for the *pilT-ftsA-ftsZ* operon in *C. perfringens* and close phylogenetic relatives. The gene encoding a protein with a COG1215 domain, which is a annotated as a glycotransferase, is positioned between the *pilT* and *ftsA* gene in some of the species.

Figure S6

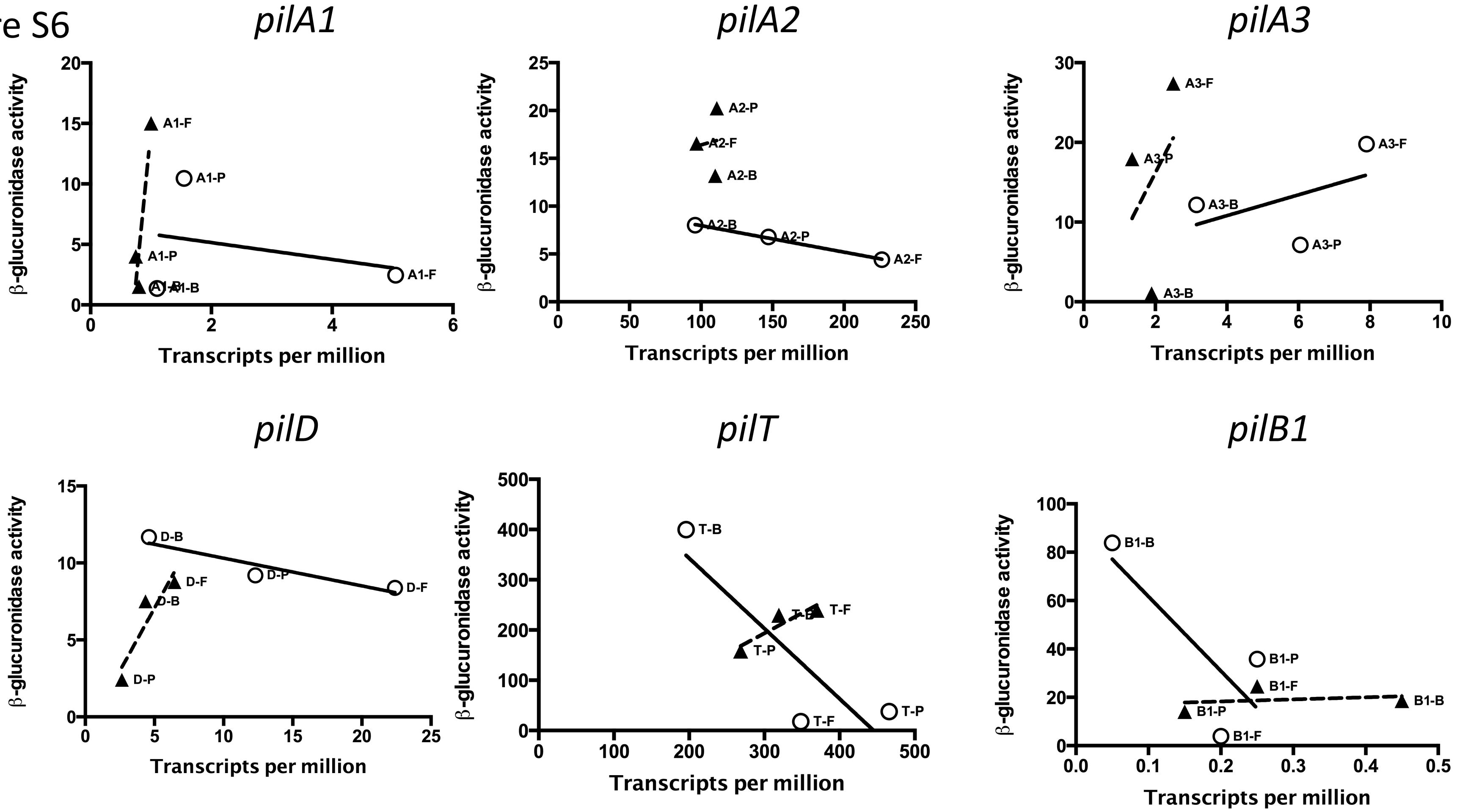

Figure S6. Plots of TPM versus β-glucuronidase activity for each promoter-*gusA* fusion. The media used were, B, BHI; P, PGY; F, FABG. The slopes for liquid grown (dashed) and plate grown (solid) are shown.

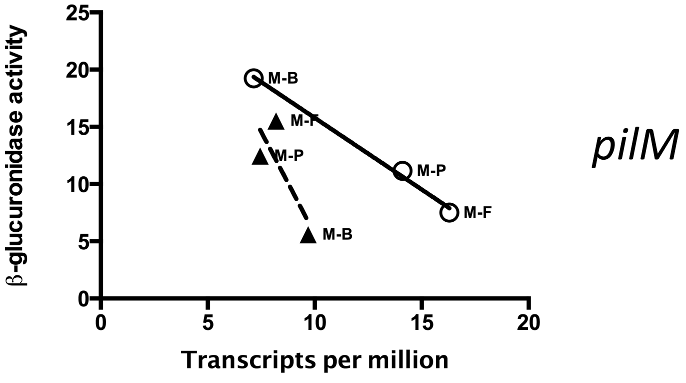

Figure S7

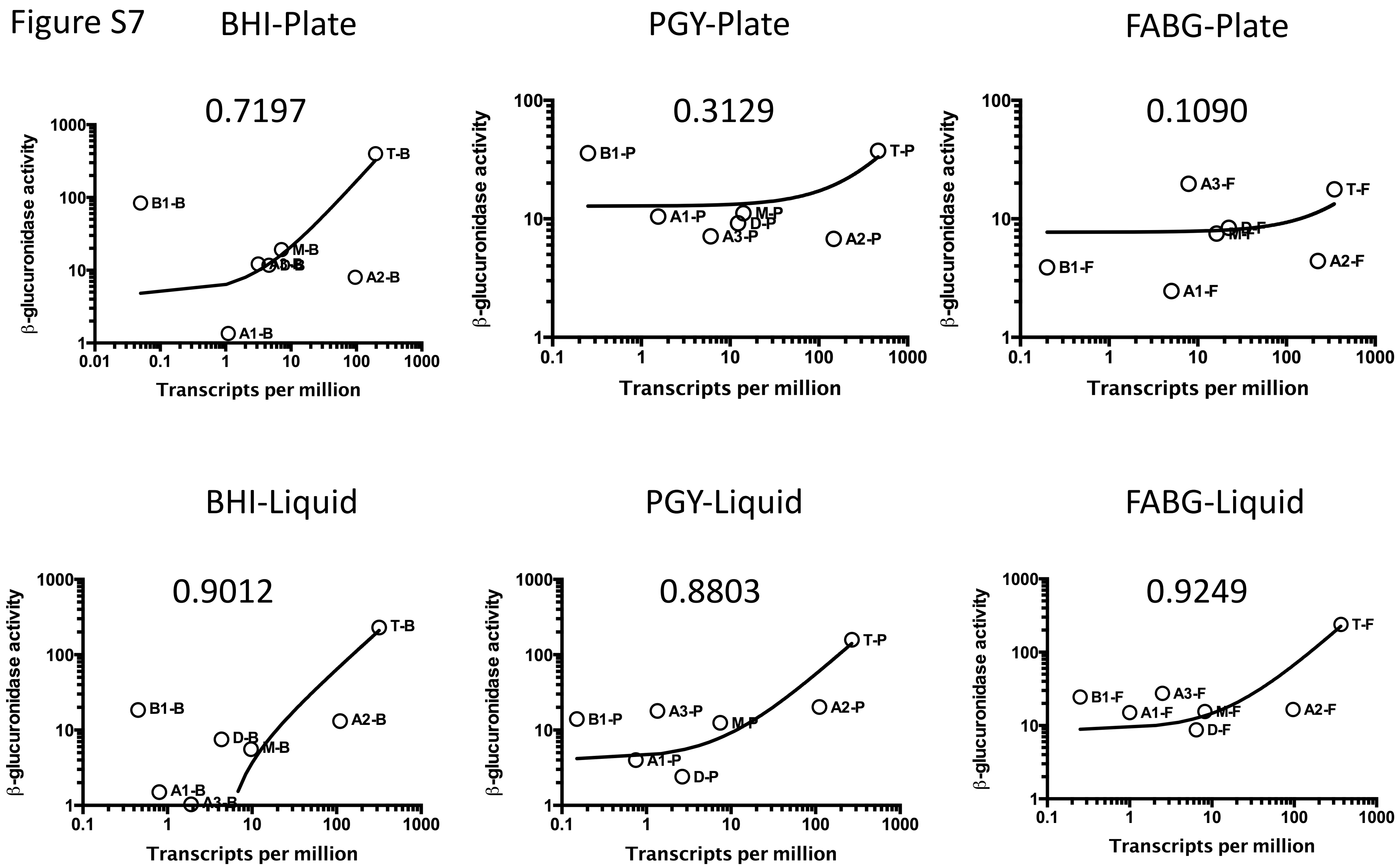

FIGURE S7. Linear regression analysis of the seven TFP promoters activity plotted versus the TPM for the corresponding gene. The growth conditions for each experiment are listed for each figure along with the R^2 value for the curve.

Figure S8

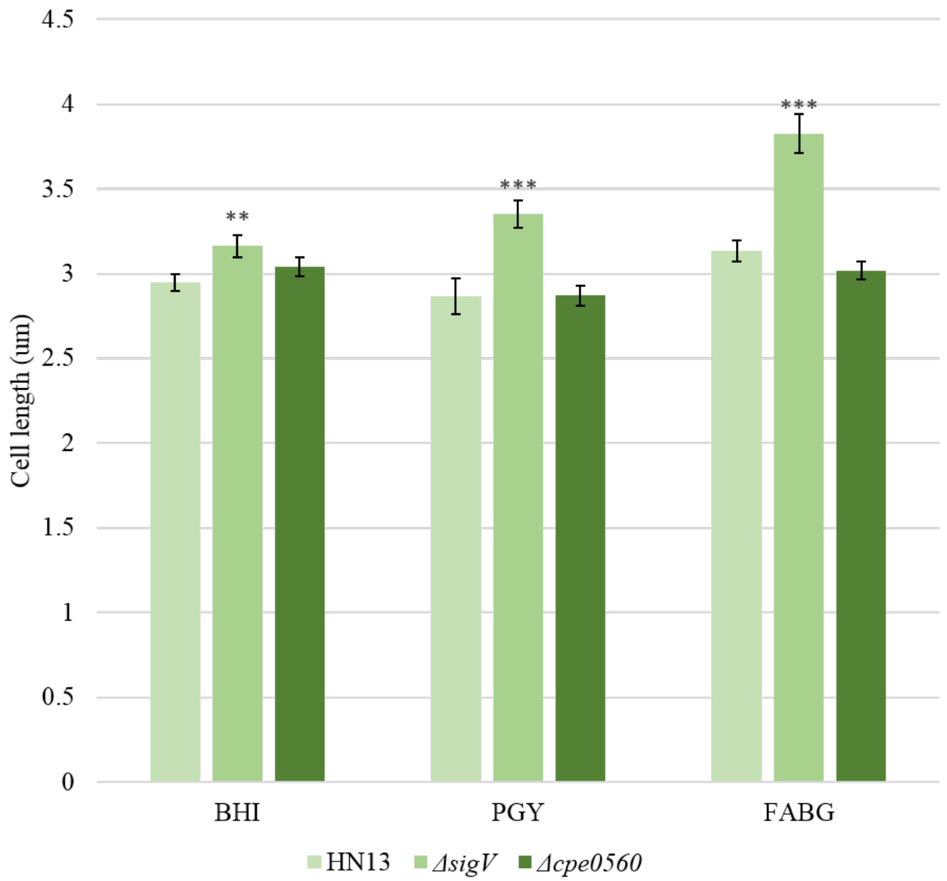

FIGURE S8. Cell lengths of HN13, Δ*sigV*, and Δ*cpe0560* cells. Bacteria were grown on plates of three media. Lengths were obtain by measuring distance between poles in ImageJ. The mean and SD for each strain and growth conditions are shown. **, P <0.01; *** P<0.001 in length in comparison to strain HN13 using the students two tailed t-test.

Figure S9

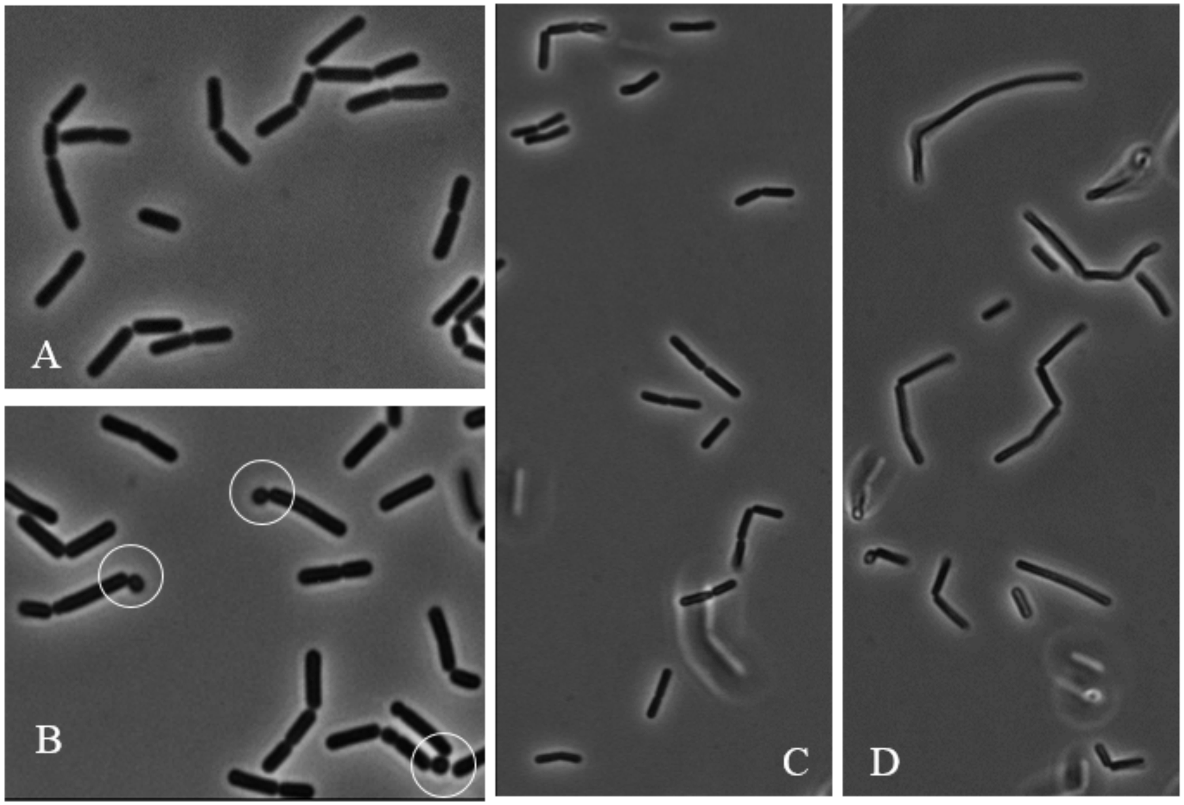

FIGURE S9. Irregular cell morphologies of Δσ^V plate-grown cells. Cells were grown at 37°C overnight, scraped from the edges of colonies, and visualized by phase microscopy. (A) HN13, PGY plate, 100x. (B) Δσ^V, PGY plate, 100x. Circles indicate minicells. (C) HN13, FABG plate, 60x. (D) Δσ^V, FABG plate, 60x.
